# Structure and Functions of Microtubule Associated Proteins Tau and MAP2c: Similarities and Differences

**DOI:** 10.3390/biom9030105

**Published:** 2019-03-16

**Authors:** Kateřina Melková, Vojtěch Zapletal, Subhash Narasimhan, Séverine Jansen, Jozef Hritz, Rostislav Škrabana, Markus Zweckstetter, Malene Ringkjøbing Jensen, Martin Blackledge, Lukáš Žídek

**Affiliations:** 1 Central European Institute of Technology, Masaryk University, Kamenice 5, 625 00 Brno, Czech Republic; katerina.melkova@ceitec.muni.cz (K.M.); vojtech.zapletal@ceitec.muni.cz (V.Z.); subhash.narasimhan@ceitec.muni.cz (S.N.); severine@chemi.muni.cz (S.J.); jozef.hritz@ceitec.muni.cz (J.H.); 2 Faculty of Science, National Centre for Biomolecular Research, Masaryk University, Kamenice 5, 625 00 Brno, Czech Republic; 3 Institute of Neuroimmunology, Slovak Academy of Sciences, Dúbravská cesta 9, 845 10 Bratislava, Slovakia; rostislav.skrabana@savba.sk; 4 Axon Neuroscience R&D Services SE, Dvořákovo nábrežie 10, 811 02 Bratislava, Slovakia; 5 German Center for Neurodegenerative Diseases (DZNE), Von-Siebold-Str. 3a, 37075 Göttingen, Germany; Markus.Zweckstetter@dzne.de; 6 Department of NMR-Based Structural Biology, Max Planck Institute for Biophysical Chemistry, Am Fassberg 11, 37077 Göttingen, Germany; 7 University Grenoble Alps, CEA, CNRS, 38000 Grenoble, France; malene.ringkjobing-jensen@ibs.fr (M.R.J.); martin.blackledge@ibs.fr (M.B.)

**Keywords:** microtubule associated protein, tau, intrinsically disordered protein, phosphorylation, nuclear magnetic resonance

## Abstract

The stability and dynamics of cytoskeleton in brain nerve cells are regulated by microtubule associated proteins (MAPs), tau and MAP2. Both proteins are intrinsically disordered and involved in multiple molecular interactions important for normal physiology and pathology of chronic neurodegenerative diseases. Nuclear magnetic resonance and cryo-electron microscopy recently revealed propensities of MAPs to form transient local structures and long-range contacts in the free state, and conformations adopted in complexes with microtubules and filamentous actin, as well as in pathological aggregates. In this paper, we compare the longest, 441-residue brain isoform of tau (tau40), and a 467-residue isoform of MAP2, known as MAP2c. For both molecules, we present transient structural motifs revealed by conformational analysis of experimental data obtained for free soluble forms of the proteins. We show that many of the short sequence motifs that exhibit transient structural features are linked to functional properties, manifested by specific interactions. The transient structural motifs can be therefore classified as molecular recognition elements of tau40 and MAP2c. Their interactions are further regulated by post-translational modifications, in particular phosphorylation. The structure-function analysis also explains differences between biological activities of tau40 and MAP2c.

## 1. Introduction

**Figure 1:**
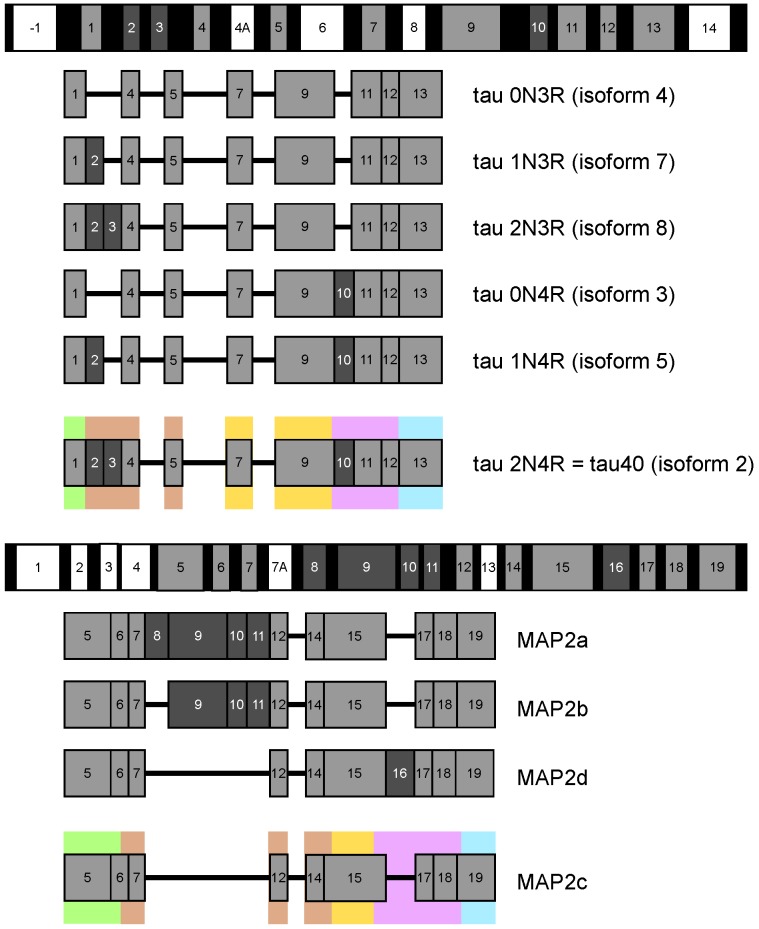
Organization of the DNA sequences of tau (top) and MAP2 (bottom) and splicing isoforms expressed in brain. Exons not expressed in brain isoforms are shown in white, exons expressed in all isoforms are shown in light gray, and exons expressed in some isoforms are shown in dark gray. Regions of isoforms discussed in this review are shown in boxes of different colors. The exons of tau and MAP2 are numbered according to Cailet-Boudin et al. [4] and Sündermann et al. [45], tau isoforms are numbered according to the National Center for Biotechnology Information (NCBI) RefSeq database.

The stability and dynamic behavior of the cytoskeleton are regulated by structural microtubule associated proteins (MAPs) [1]. Major structural MAPs in brain nerve cells are the MAP2 and tau protein [2]. Polypeptide chains of MAP2 and tau have a bipolar character; their microtubule binding repeats (MTBRs) and projection domain (PD) lie at C- and N-terminal part of molecule, respectively. Both proteins exist as multiple alternatively spliced isoforms (Figure 1), differing in the presence of the second MTBR (exon 10 of tau and exon 16 of MAP2) and of several exons in PD (including long exon 9 distinguishing high-molecular weight isoforms of MAP2). Expression of individual isoforms is developmentally and regionally regulated. Most notably, adult neurons express high-molecular weight MAP2 isoforms specifically in cell bodies and dendrites [2], whereas the six tau protein isoforms are found predominantly in axons [3]. Low molecular weight MAP2 isoforms are expressed in developing neurons, mostly prenatally, whereas high-molecular weight tau isoforms comprising exons 4a and 6 are expressed only in peripheral tissues [4].

Both high- and low-molecular weight isoforms of the discussed MAPs have been studied extensively. However, detailed and residue-specific description of transient structural features is currently available only for MAP2c and for the tau isoforms presented in Figure 1. Although the structure-function relationship is not described for MAP2a and MAP2b in such details, it is evident that high-molecular weight isoforms of MAP2 play important and specific biological roles. The increased expression of MAP2a (and decreased expression of MAP2c) correlates with the reduction of cytoskeleton dynamics during neuronal maturation [5]. Presence or absence of long PD seems to be one of the factors (together with messenger RNA (mRNA) compartmentalization) controlling cellular localization of MAP2 isoforms [2,5]. Transfection experiments suggested that the presence of long PD prevents MAP2a/b from entering the axons [6]. The size of PD also influences properties of microtubule (MT) bundles induced by MAP2 isoforms, with a potential impact on the kinesin- and dynein-dependent transport along microtubules [2]. It has been suggested that the length of PD of MAP2 and tau isoforms regulates the spacing of MTs inside MT bundles in dendrites and axons [7]. Experiments with tau adsorbed on mica surface (a proxy for the MTs) suggested that the MT distance spacer may be formed by an antiparallel charge-dependent dimerization of PDs from opposing tau molecules [8]. Direct measurement of forces between tau-coated MTs at physiological, sub-stoichiometric tau:tubulin ratio revealed that a structure of the PD layer may depend on both the amount of bound tau and the molecular crowding, where the isoforms with longer PD conferred a much higher resistance to MT bundling under increased osmotic pressure [9]. Interestingly, MAP2 and tau isoforms may bind and crosslink MTs with F-actin by partially overlapping multiple interaction sites in the MTBRs [10,11]. The apparent binding affinity of tau to F-actin is comparable to its affinity to MTs [12]. Mice with either MAP2 or tau gene knockout were apparently normal, but simultaneous disruption of MAP1b gene led to a high prenatal mortality [13,14,15]. It suggested an important role of MAP2/tau in neurogenesis, which can be (partially) rescued by MAP1b.

Despite the similarity in overall molecular organization and physiological function, along with a high sequence identity of the MTBRs, MAP2 and tau have contrasting significance in pathophysiological processes resulting in chronic diseases. The involvement of MAP2 in pathogenesis (based on detection of high-molecular weight isoforms in adult tissues) is relatively modest. Depletion of MAP2 has been associated with a Lewy body variant of Alzheimer’s disease [16], whereas colocalization of MAP2 with α-synuclein in Lewy bodies was shown in Parkinson’s disease [17]. Disappearance of dendritic high-molecular weight MAP2 isoforms was recently described in striatum of patients with Huntington’s disease [18]. 3β-methoxy-pregnenolone, which binds to MAP2 isoforms in vitro and increases its ability to stimulate tubulin assembly, has antidepressant efficacy in rats [19]. In contrary, tau protein has been long known for its involvement in various neurodegenerative illnesses. Tau was discovered as the constituent of neurofibrillary pathology in Alzheimer’s disease and other tauopathies, comprised of more than 20 individual disorders; for review see [3,20]. Tens of exonic and intronic mutations in tau gene with direct implications for neuropathological conditions were described. As the extent of tau pathology correlates with the disease progression, tau has become an appealing target for various therapeutic strategies including small-molecule inhibition of tau aggregation and phosphorylation, anti-sense oligonucleotide therapy, passive and active immunotherapy [21,22,23]. Pathological forms of tau include high-molecular weight oligomers and polymers, in which the tau molecules associate via their MTBRs, forming tightly ordered amyloid structures [20,24]. The fact that MAP2 was never found polymerized in vivo despite 90% sequence homology of respective MTBRs, aggregation properties of chimeric proteins, and site-directed mutagenesis indicate a key role of individual tau residues for initiation and propagation of polymerization [25,26].

Isoforms of MAP2c and tau are subjects to frequent post-translational modifications [27]. Modifications at specific sites regulate binding to MTs and MT dynamics [28,29,30,31]. Importantly, MAP2 and tau isoforms have a highly disordered polypeptide backbone (see below), which can be selectively modulated by protein phosphorylation. Apart from introducing two negative charges per phosphate group (at physiological pH), which can influence the long-range contacts, phosphate oxygens may form specific local interactions with neighbouring main chain polar groups (carbonyl, amine) [32,33], changing the distribution of preferred transient conformations in the conformational ensemble. For instance, using short tau phosphopeptides it has been shown that phosphorylation of proline-rich regions of tau may induce local conformational changes to polyproline II helix [31,34]. Physiological phosphorylation of MAP2 exceeds that of tau (1–2.5 and 0.5–1 phosphates per 100 residues in MAP2 and tau, respectively). However, phosphorylation of tau increases four-fold under pathological conditions and has been associated with tau toxic gain of function [3,28]. In developing brain both proteins exhibit elevated phosphate content, whereas in adulthood the overall phosphorylation decreases. Kinases phosphorylating tau and MAP2 isoforms have been reviewed previously [5,35,36,37]. Tyrosine kinases include Fyn, Syk, c-Abl, Arg. The list of serine/threonine kinases is long, those discussed as examples in this paper are listed in Table 1.

Both tau and MAP2 isoforms are intrinsically disordered in the free state and interact with complex cytoskeletal structures. It makes them challenging targets for structural studies. Nevertheless, improved resolution of nuclear magnetic resonance (NMR) spectroscopy and electron microscopy (EM) made detailed studies of free and interacting MAPs possible. Structural features of highly flexible free tau40 [38,39] and MAP2c [40] isoforms have been described using liquid-state NMR, complemented by quantitative conformational analysis. Recently published atomic and near-atomic resolution data allow us to look for molecular mechanisms of physiological processes altered in chronic diseases. Combination of cryo-electron microscopy (cryo-EM), NMR, and computational analysis provided reliable high-resolution models of tau interacting with microtubules (MTs) [41,42,43], actin filaments [10], and forming filaments in brains of patients suffering from the Alzheimer’s disease [24,44]. Our goal is not to provide here a complete list of physiological and pathological roles of the reviewed proteins or a summary of all experimental structural data. Instead, we discuss (i) how molecular functions and dysfunctions of tau and MAP2 can be traced to sequence motifs forming transient, but well defined local structures with distinct dynamics; and (ii) how differences in such motifs explain functional diversity of tau and MAP2. Specifically, we limit the discussion to the longest, 441-residue brain isoform of human tau (clone htau40, splicing variant 2N4R), and the shortest, 467-residue isoform of rat MAP2 (94% sequence identity with the corresponding human isoform), referred to as tau40 and MAP2c in this paper, respectively. Residue numbering is referred to these two isoforms as well. Our selection of these MAPs mostly reflects the amount of available experimental data reported in the literature. Also, the similar lengths of the chosen isoforms simplify direct comparison of both proteins.

## 2. Measurement and Presentation of Transient Local Structures

As mentioned above, our goal is to relate the biological functions of tau40 and MAP2c to transient secondary structures observable in a free state. Therefore, we start our discussion by briefly commenting how the structural data were obtained, and how they are presented in this paper.

So-called intrinsically disordered proteins (IDPs), including tau40 and MAP2c, do not adopt a random conformation as many synthetic polymers. Instead, they exist in multiple rapidly inter-converting structures defined by the same interactions as well-ordered proteins. Therefore, transient secondary structure motifs and long-range order are present in IDP samples, and their populations can be estimated by analyzing experimental data.

Formation of transient local structures discussed in this paper was inferred from NMR chemical shifts, measured in several studies [38,40,46,47,48,49,50,51,52,53,54,55], and converted to populations of conformers occupying different regions of the Ramachandran diagram by the ASTEROIDS algorithm [39,56,57]. The ASTEROIDS analysis also included results of NMR paramagnetic relaxation enhancements and small-angle X-ray scattering, describing long-range contacts and overall shapes of the studied molecules, respectively. Technically, the result of the analysis was a set of three-dimensional structures selected to match the experimental data. This allowed us to statistically evaluate not only populations of backbone torsion angles of individual amino acids, but also occurrence of their specific combinations, which give rise to secondary structure elements (Figures S1–S4 in Supplementary Materials). Distribution of these parameters are presented in this paper as an experimental measure of the propensity to form such structural motifs.

Although full-length tau40 and MAP2c can be studied by current NMR techniques, analysis of shorter fragments provides useful information about the influence of long-range contacts on local structure. Based on a comparison of ${}^{13}$C${}^{\alpha }$ chemical shifts, secondary structures of full-length tau40 and of three shorter constructs are very similar [38] and similar results were obtained also for MAP2c and its fragments (Figure S5 in Supplementary Materials). This similarity underlines the fact that secondary structure propensities are highly specific properties of the sequence motifs. However, it should be stressed that although long-range contacts do not change the overall statistics of secondary structures in free tau40 and MAP2c, they define distinct global (“tertiary”) structures. External factors such as interactions with binding partners or molecular truncation [58] may result in conformational selection of minor, but functionally important global states. For example, interaction of tau MTBRs with the monoclonal antibody DC8E8 was enhanced up to 25 times after removal of N- and C-termini of the molecule [59].

**Figure 2:**
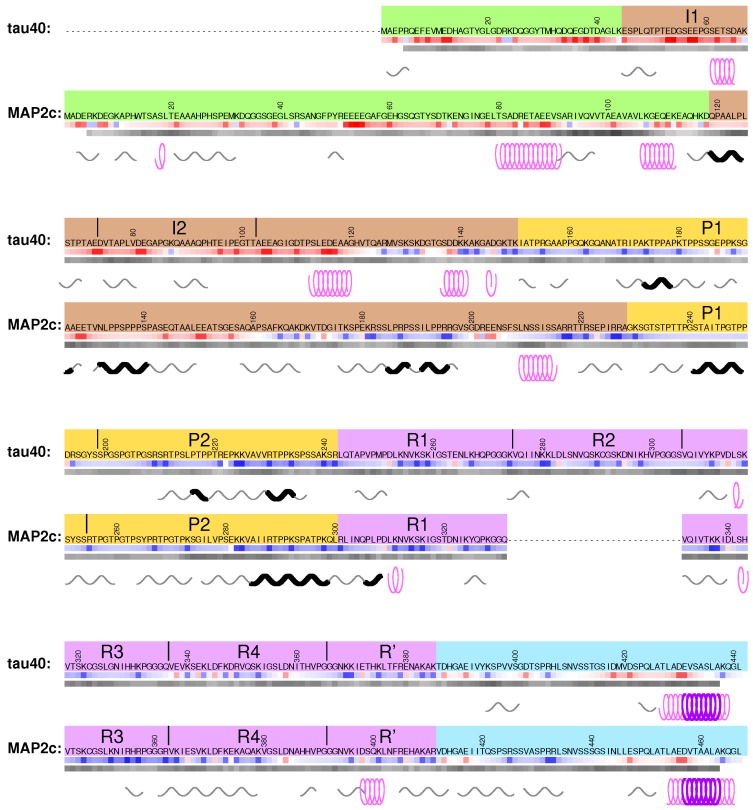
Sequences of human tau40 and rat MAP2c shown with backgrounds distinguishing individual regions. The pale green background indicates the N-terminal region, the brown background indicates the variable central region preceding the proline-rich domains, the yellow background indicates the proline-rich domains, the violet background indicates MT binding domains, and the cyan background indicates the C-terminal region. Charge distribution, hydropathicity, and preferred secondary structures are shown below the sequences. The charge distribution is represented by the color in the upper rows of squares below the sequences, corresponding to a relative electrostatic potential approximated by $\sum_{j}CQ_{i}/(d_{0}+d_{1}|n_{i}-n_{j}\left|\right)$, where $Q_{i}$ and $n_{i}$ are charge and sequential number of the *i*-th residue, *C* is a constant including the electric permittivity, and $d_{k}$ are distance constants. The ratio $d_{1}/d_{0}$ was set to 2.0 and the colors were chosen so that red and blue correspond to the highest negative and positive potential, respectively, which makes the color code independent of $C/d_{0}$ [40]. The hydropathicity index according to Kyte and Doolittle [61] is shown as darkness of the lower rows of squares below the sequences (white and black correspond to the values of −4.5 and +4.5, respectively). Formation of transient α-helices is shown as pink and purple symbols (corresponding to more than 5% and 15%, respectively, of structures with continuous stretches of four amino acids in α-helical conformation in the ensembles selected by the ASTEROIDS analysis of NMR chemical shifts [39,57]), formation of transient polyproline II structures is shown as gray and black symbols (corresponding to more than 5% and 15%, respectively, of structures with continuous stretches of four amino acids in polyproline II conformation in the ensembles selected by the ASTEROIDS analysis).

**Figure 3:**
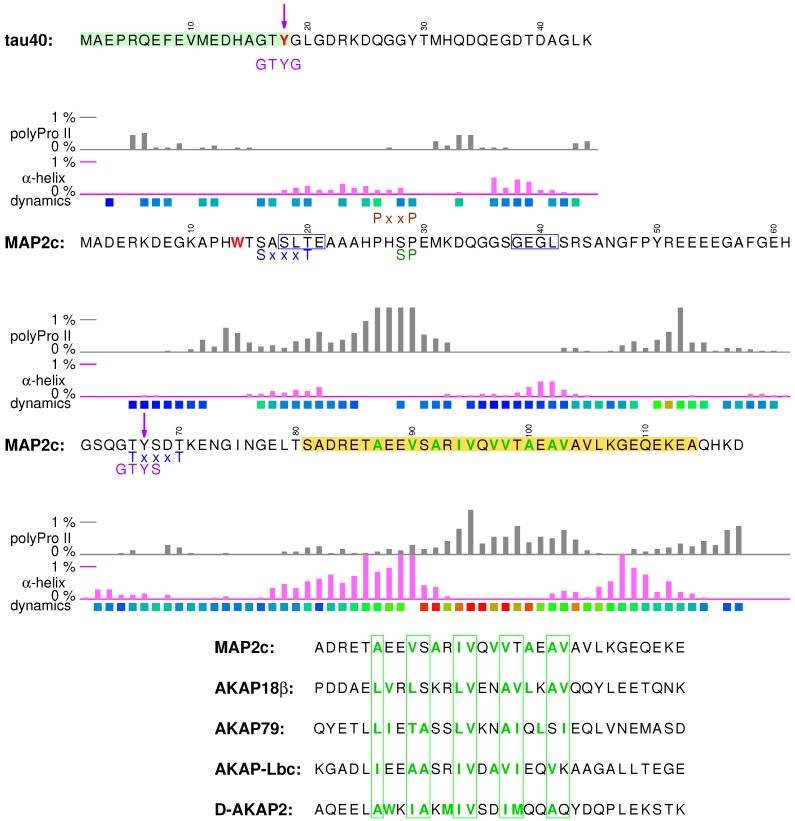
N-terminal sequences of human tau40 and rat MAP2c. The pale green and yellow boxes indicate the region of tau interacting with membranes and the region of MAP2c interacting with the regulatory RII subunit of PKA, respectively. Highly conserved residues are shown in red. Hydrophobic residues in the region interacting with the regulatory RII subunit of PKA are shown in green. Phosphorylated tyrosines are marked with purple arrows. Minimal SH3 interaction motifs (PxxP) are shown in brown above the sequences. Recognition motifs of proline-directed kinases (green) and GSK3β (blue), and Fyn phosphorylation motifs (purple) are shown below the sequences. Populations of continuous stretches of seven amino acids in the α-helical and polyproline II conformations in the ensembles of structures selected by the ASTEROIDS analyses based on measured NMR chemical shifts [31,57] are shown as pink and gray bars placed in the middle of the stretches, respectively. Residues forming β-turns are shown in blue frames. Dynamics of individual amino acids, measured as transverse NMR relaxation rate, is described by colors of the boxes below the secondary structure symbols (blue corresponding to flexible residues with the relaxation rate of 2 s${}^{-1}$ or lower, red corresponding to the most ordered residues with the relaxation rate of 10 s${}^{-1}$ or higher). The presented values are the relaxation rates measured at 700 MHz and 5 ${}^{\circ }$C for tau40 [38] and values recalculated from relaxation rates measured at 950 MHz and 27 ${}^{\circ }$C for MAP2c [57] in order to account for the magnetic field difference. No temperature correction was applied because relaxation rates of IDPs cannot be easily recalculated for a different temperature. Alignment with the sequences of well-ordered AKAPs is shown under the region interacting with the regulatory RII subunit of PKA (preferred positions of hydrophobic residues in the amphiphilic binding α-helices are shown in green frames).

**Figure 4:**
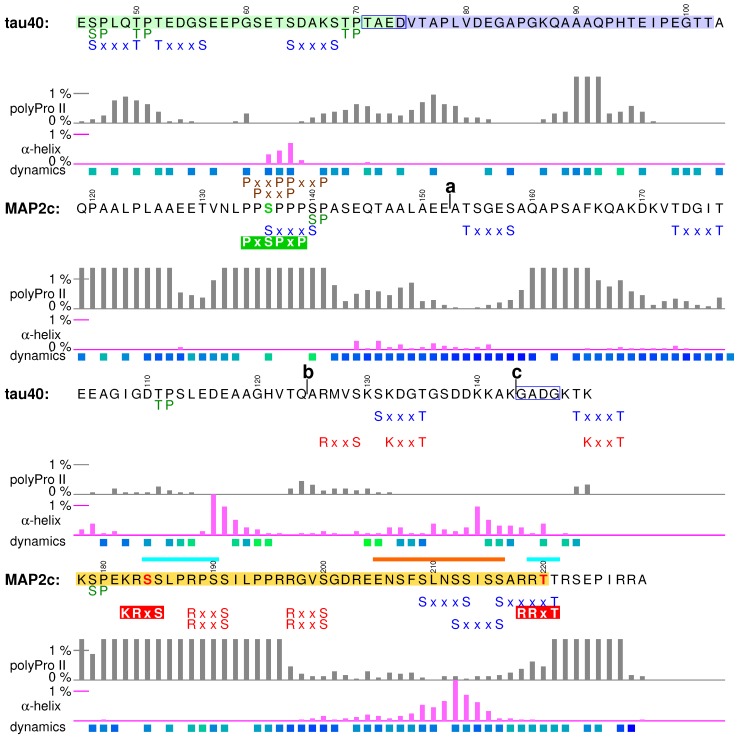
Sequences of variable central regions preceding the proline-rich domains of human tau40 and rat MAP2c. Tau inserts I1 and I2 are shown in pale green and pale blue boxes, respectively. The motif in the yellow box is described in the text. PKA recognition motifs and residues rapidly phosphorylated by PKA in vitro are shown in red. Interactions of MAP2c with plectin and 14-3-3 proteins are shown as orange and cyan bars above the sequence. Letters **a**, **b**, and **c** show position of long exons in the high-molecular weight tau and MAP2 isoforms. Other symbols are used as described for Figure 3.

**Figure 5:**
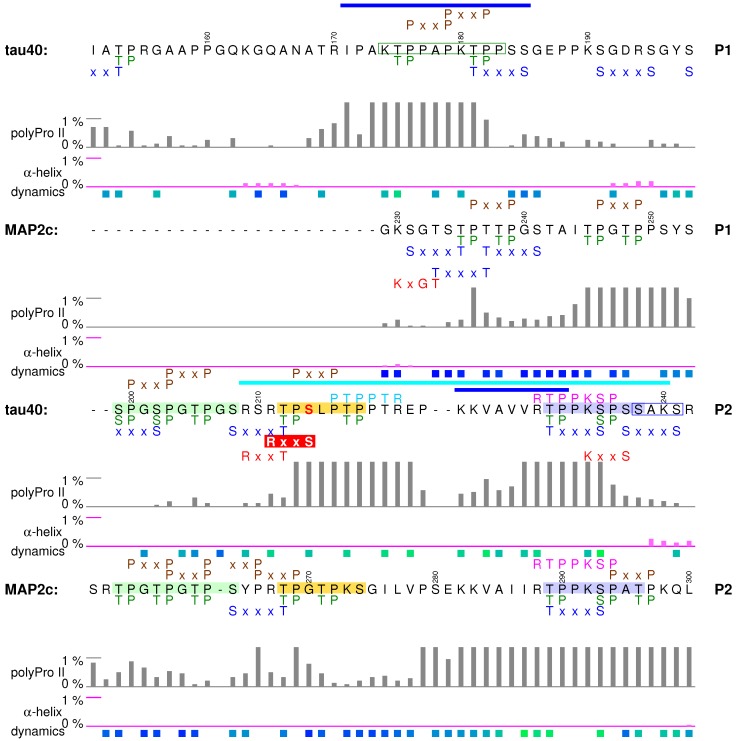
Sequences of proline-rich domains of human tau40 and rat MAP2c. Complex phosphorylation sites shown in the green frame and in pale green, pale blue, and red boxes are described in the text. The classical Class I and Class II SH3 binding sites are presented in magenta and cyan, respectively, above the sequence. Regions of tau interacting with tubulin are indicated by the blue bars above the sequence. Other symbols are used as described for Figure 3 and Figure 4.

**Figure 6:**
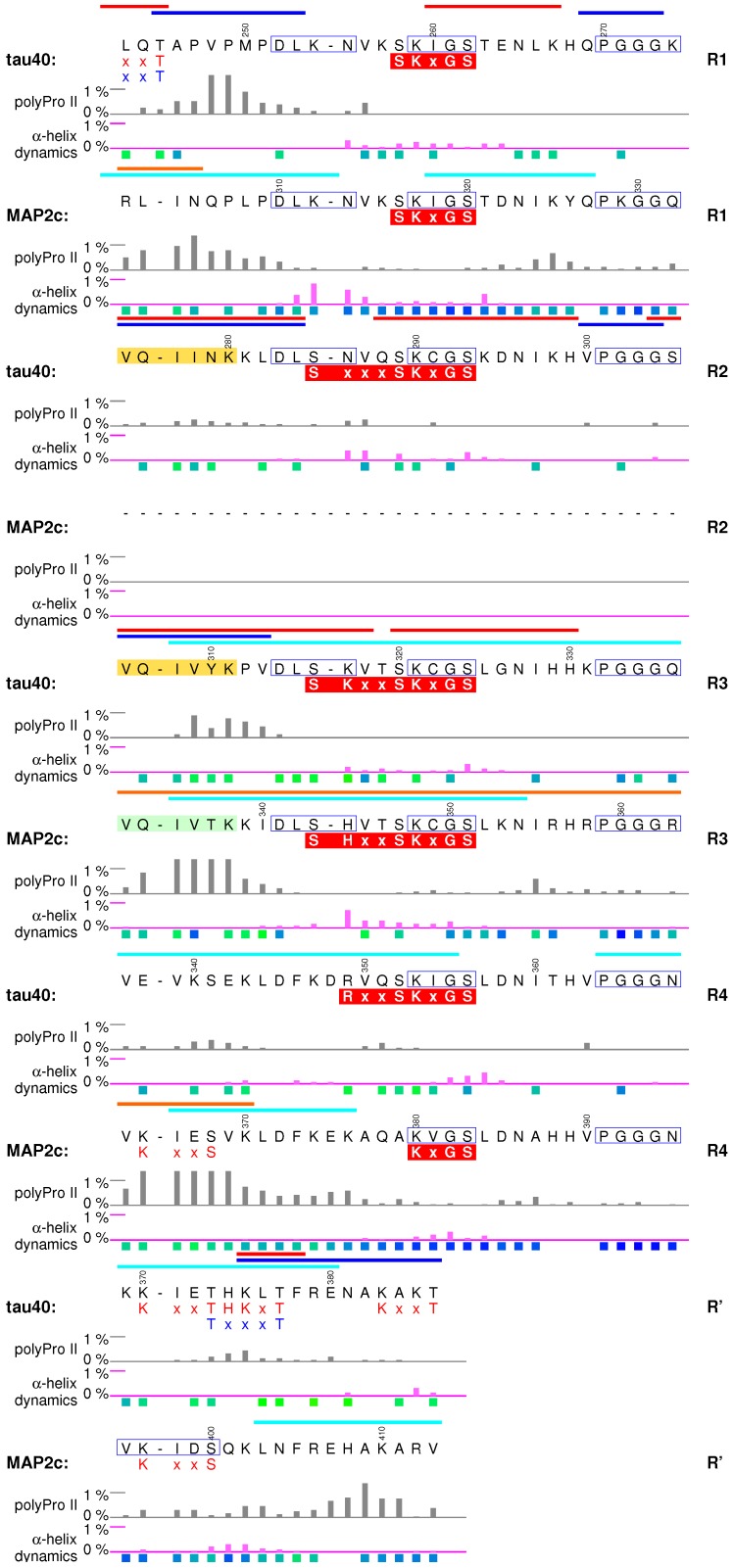
MTBDs of human tau40 and rat MAP2c. MTBRs 1–4 are labeled R1–R4, respectively, the following sequence homologous with the N-terminal sequence of MTBRs is labeled R${}^{\prime }$. Aggregation sites of tau40 are shown in yellow boxes and the corresponding sequence of MAP2c is shown in a pale green box. Complex phosphorylation sites including the MARK recognition motifs are shown in red boxes. Regions of tau interacting with filamentous actin are indicated by the horizontal red bars above the sequence. Other symbols are used as described for Figure 3 and Figure 4.

**Figure 7:**
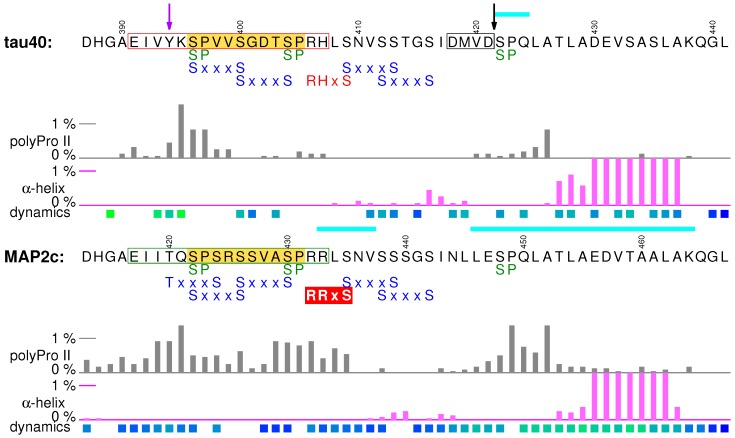
C-terminal sequences of human tau40 and rat MAP2c. The region of tau interacting with cholinergic muscarinic receptors M1 and M3 is shown in the red frame, and the corresponding region of MAP2c is shown in the green frame. The PHF-1 epitope of tau40 is shown in the yellow box and the MAP2c site phosphorylated most rapidly by PKA is shown in the red box. The black frame and the black arrow mark the caspase-3 recognition sequence and cleavage site, respectively. Other symbols are used as described for Figure 3 and Figure 4.

Visualization of the local structures is not trivial because multiple locally ordered structural motifs cannot be aligned in a single three-dimensional structural model. Therefore, in this paper we discuss conformations of tau40 and MAP2c without showing the three-dimensional structures explicitly. Occurrence of two secondary structure motifs that were present most often in the ensembles of conformers selected by the ASTEROIDS analysis (α-helix and polyproline II) is schematically depicted in Figure 2. To give more quantitative description of the propensity to form secondary structures, populations of continuous stretches of seven residues found in the same conformation in the selected ensembles are plotted for individual tau40 and MAP2c regions in Figure 3, Figure 4, Figure 5, Figure 6 and Figure 7 [57]. Residues with increased populations of β-turn conformations [60] are also marked in Figure 3, Figure 4, Figure 5, Figure 6 and Figure 7.

## 3. N-Terminal Regions

The sequences of tau40 and MAP2c start with PDs, involved in the regulation of microtubule spacing. N-terminal regions of PDs (pale green boxes in Figure 1 and Figure 2) are rich in acidic and hydrophobic amino acids. As the N-terminal regions of tau and MAP2 differ in size, structural properties, and physiological functions, structural characteristics and examples of biological roles associated with these regions are discussed separately for tau40 and MAP2c in this section.

### 3.1. Structural Properties of N-Terminal Region of tau40

In the free form, the N-terminal region of tau exhibits low content of secondary structure, but makes transient contacts with the positively charged proline-rich regions and strongly helical C-terminus [38,39,62]. These intramolecular electrostatic interactions contribute to formation of bent “paper-clip” tertiary structure of tau (together with contacts between MTBRs and the C-terminal region) [62], and to consequent functional links between distant regions. The intramolecular contacts also compete with intermolecular interactions. In the following paragraphs, we present biological roles that seem to depend on balance between intra- and intermolecular interactions of functionally important sequence at the very N-terminus of tau.

### 3.2. Phosphatase Activation by Tau N-Terminus and Axonal Transport

The first role of tau discussed in this section is modulation of the axonal transport, closely related to the localization of tau in axones. The motif shown in the pale green box in Figure 3 was described as the phosphatase-activating domain (PAD) because a peptide consisting of amino acids Ala2–Tyr18 activates a signaling cascade involving protein phosphatase 1 (PP1) and GSK3β kinase [63,64]. Phosphatase-activating domain contains an imperfect consensus PP1 binding motif ${}_{5}$RQEF${}_{8}$ [65], which explains why Tau specifically interacts with PP1 and targets PP1 to MTs. The PP1 converts GSK3β to its active (dephosphorylated) form, which phosphorylates kinesin light chain. This inhibits fast axonal transport mediated by kinesin moving along MTs since phosphorylation of the kinesin releases the kinesin-bound cargo. It was suggested that a lack of the native intramolecular contacts in aggregates and other pathological forms of tau lead to axonal transport dysfunction accompanying Alzheimer’s disease and other neurodegenerative diseases. In the absence of the native contacts, PAD is more exposed, the PP1-GSK3β pathway is hyperactivated, and the axonal transport is inhibited.

The N-terminal region of tau also represents an excellent example of regulation of biologically important interactions by post-translational modifications. The last amino acid of PAD is highly conserved Tyr18 and its neighboring residues match the ideal recognition sequence of the Fyn kinase (GTYG, preceded by an acidic and an aromatic residue) identified by a phage display [66]. Tyr18 is indeed preferentially phosphorylated by several kinases including Fyn [36,67]. It has been shown that the phosphomimetic mutation Y18E prevents the inhibition of axonal transport by PAD. Based on this finding, it was proposed that phosphorylation of Tyr18 regulates the cargo delivery [68].

The N-terminus of tau also binds another protein involved in axonal transport, dynactin. Dynactin is a multi-protein complex essential for axonal transport, playing an important role in mediating the binding of the MT motor dynein to its membranous cargoes [69,70]. The dynactin complex forms an actin-like filament and a lateral arm able to interact with MTs. The major component of the arm is p150. Its N-terminal domain binds MTs, while C-terminal 230 amino acids contribute to a structure, called shoulder, sitting on the filament. It has been reported that tau sequences encoded by exons 1 and 4 interact independently with the C-terminus of p150 and stabilize MT binding to the dynactin complex [71]. Interaction with exon 1 is affected by mutation of Arg5, associated with frontotemporal dementia and parkinsonism linked to chromosome 17 (FTDP-17). Therefore, PAD is most likely involved in the binding and mutations in PAD can cause defects in axonal transport.

### 3.3. Interactions of Tau with Neuronal Membranes

Another physiological role regulated by phosphorylation of Tyr18 is tau’s interaction with neuronal membranes [72]. It has been shown that phosphorylated Tyr18 interacts with the Src-homology 2 (SH2) domain of the tyrosine kinase Fyn, and that Fyn-mediated phosphorylation induces trafficking of tau to detergent-resistant membrane microdomains in mouse primary cortical neurons [73]. These membrane microdomains are involved in intracellular signaling and therefore regulation of the Tyr18 phosphorylation may be an important factor in keeping normal physiological conditions. Furthermore, tau is recruited to the lipid rafts and phosphorylated at Tyr18 when SHSY-5Y cells are treated with the Aβ peptide [74]. It indicates that Tyr18 phosphorylation and interactions of tau with membranes may play a role in the Aβ-induced neurotoxicity.

### 3.4. Structural Properties of N-Terminal Region of MAP2c

Physiologically important structural features of the N-terminal region of free MAP2c are more complex than those described for tau40. Occurrence of turns (Figure 3) between more extended segments suggests formation of transient hairpin structures. NMR relaxation [57] suggests that aromatic amino acids, frequent among the first 80 residues of MAP2c (most notably Trp14, Phe48, and Tyr50), are parts of a more ordered structure that is not observed in tau40 [38] and that includes also the following segment (Ser81–Glu113), where all hydrophobic residues are aliphatic. Similar to tau40, the N-terminal region of free MAP2c interacts with the proline-rich and MT-binding domains [57]. Unlike in tau40, a very distinct secondary structure motif is present in the N-terminal region of MAP2c. Residues Ser81–Glu113 exhibit a strong propensity to form an α-helix interrupted by a more extended segment. Biological relevance of this motif is discussed in Section 3.6.

### 3.5. Neurosteroid Binding to the N-Terminal Region of MAP2c

Neural activities are regulated by steroid compounds (neurosteroids) synthesized directly in the brain, independently of the peripheral endocrine glands [75]. Neurosteroids specifically bind to MAP2 isoforms and stimulate MT polymerization and consequently neurite extension [76]. Experiments with truncated MAP2c suggested that the N-terminal region is essential for binding [77,78]. Our unpublished NMR data confirm that neurosteroids bind to the N-terminal region, but presence of C-terminal regions is needed too (Figure S6 in Supplementary Materials). This is consistent with the NMR relaxation data, showing formation of a more ordered structure, and with a homology model predicting residues Met1–Lys71 to form the major portion of a binding pocket [77,78] for neurosteroids. Therefore, the N-terminal region of MAP2c can be viewed as a transiently structured receptor of steroids controlling neurite extension and thus affecting neuronal plasticity.

### 3.6. Binding Site for the RII Regulatory Subunit of PKA in the N-Terminal Region of MAP2c

Residues Ser81–Glu113 (encoded by C-terminus of exon 5 and N-terminal half of exon 6, yellow box in Figure 3) represent a very characteristic structural and functional motif of MAP2c, a binding site for the RII regulatory subunit of PKA. The dimeric RI and RII PKA subunits recognize long amphiphilic α-helices (at least five turns) of well-folded A-kinase anchoring proteins (AKAPs, see sequence comparison with MAP2c in Figure 3) [79]. As mentioned above, the terminal parts of the sequence Ser81–Glu113, ${}_{81}$SADRETAEEVSA${}_{92}$ and ${}_{107}$KGEQEKE${}_{113}$, have a high propensity to form α-helix. However, the middle part of the binding site is extended, with increased propensity to form polyproline II conformation. Remarkably, the middle region is most rigid, as revealed by NMR relaxation [57]. The second α-helix of the RII-binding motif also contributes to the proposed steroid binding pocket. The regions interacting with the RII subunit of PKA and with neurosteroids thus form one structural unit, most ordered in the whole molecule, and presumably stabilized by hydrophobic interactions of aromatic residues clustered in the N-terminal region of MAP2c.

### 3.7. Phosphorylation of MAP2c Tyr67 and SH2 Binding

The N-terminal region of MAP2 isoforms is also a target of post-translational modification. Tyr67 is present in a sequence resembling the Fyn recognition motif [66] (like Tyr18 of tau), and it is the only tyrosine phosphorylated by Fyn in vitro. It is therefore likely that Tyr67 of MAP2 isoforms is an important regulatory point. For example, Tyr67 of human MAP2c is present in the SH2 binding motif pYxN, recognized by the adaptor protein Grb2 [80]. Grb2 links tyrosine kinases with the Ras signaling pathway and it has been postulated that phosphorylation of Tyr67 followed by recruitment of Grb2 are steps of an intracellular signaling cascade in fetal human brains [80]. Furthermore, Grb2 is overexpressed in Alzheimer’s disease brains and it was reported to partially revert pathological disassembly of the cytoskeleton [81]. The overlap of the target site of Tyr kinase Fyn and the binding site of a subunit regulating Ser/Thr kinase PKA with the proposed neurosteroid receptor region suggests a possible functional connection of phosphorylation cascades and steroid signaling.

### 3.8. Summary

Acidic N-terminal regions in both proteins are important for functionally relevant intramolecular tertiary contacts. Moreover, they contain important interaction sites, different in tau40 and MAP2c. The N-terminus of tau interacts with the membranes and inhibits axonal transport. The N-terminal region of MAP2c is ordered to much larger extent than that of tau40, contains a binding motif for the regulatory subunit RII of PKA, and constitutes a large portion of a neurosteroid binding site. The biological activities of the N-terminal regions are regulated by tyrosine phosphorylation.

## 4. Variable Central Regions Preceding Proline-Rich Domains

Different tau and MAP2 isoforms greatly vary in the length of the sequence between N-terminal and proline-rich domains (brown boxes in Figure 1 and Figure 2), called *variable central region* in this paper. As mentioned above, the resulting variability in the size of PDs seems to allow the nerve cells to regulate spacing of MTs. Features of these parts of MAPs are discussed below separately for tau and MAP2 isoforms.

### 4.1. Structural Properties of Variable Central Region of tau40

All tau isoforms contain relatively short stretches encoded by exons 4 and 5, but vary in the expression of two 29-amino acid long inserts, encoded by exons 2 and 3, and labeled I1 and I2, respectively (pale green and pale blue boxes in Figure 4, respectively). Both I1 and I2 are present in the longest brain isoform discussed here. I1 and I2 are highly negatively charged and prefer polyproline II conformation, with an exception of a proline-free segment ${}_{60}$GSETSD${}_{65}$ forming transient α-helix. Exon 4 encodes a flexible sequence Glu104–Pro112, connected to a transient α-helix formed by residues Ser113–Val122. In brain variants of tau, the sequence continues by short exon 5, containing a transient helix Ser137–Lys143. In the high-molecular weight isoforms of tau, not expressed in the central nervous system, exon 5 is preceded and followed by long inserts encoded by exons 4a and 6, respectively.

### 4.2. Regulatory Post-Translational Modifications: Phosphorylation of Tau Insert I1 and Truncation

Little is known about physiological function(s) of the variable central region of tau40. Nevertheless, presence of phosphorylation sites suggests possible regulatory role(s). For example, I1 of human tau contains three recognition sites of proline-directed Ser/Thr kinases (shown in green in Figure 4). Ser46 and Thr50 are phosphorylated by ERK1/2 and GSK-3β kinases in vitro [82,83,84]. Importantly, Ser46 and Thr50 are, together with Ser202, the only sites phosphorylated solely in the interphase in a Chinese hamster ovary cell line transfected by human tau40 [82]. SAPK4/p38δ is the major kinase phosphorylating Thr50, when cells are exposed to osmotic stress and the phosphomimicking mutation T50E increases the ability of tau to promote tubulin polymerization [85]. Remarkably, the residue corresponding to Thr50 of human tau40 is present in tau of primates, goats and cows, but not in tau of species not forming tau filaments, such as rodents [85]. Phosphorylated Ser46 and Thr50 were identified in Alzheimer’s disease and progressive supranuclear palsy brains [86]. It has been proposed that p38δ-mediated phosphorylation of Thr50 helps neurons to respond to the osmotic shock by stabilizing MTs, but a lack of control of the involved kinases and/or phosphatases may result in hyperphosphorylation with consequent pathological effects.

Another post-translational modification affecting biological activity of tau is truncation in the variable central region. Several truncation sites have been found in situ under physiological [87] or pathological [88] conditions. Some truncations affect biological activity of tau. A tau40-derived fragment beginning at Gln124 exhibited enhanced stabilization of MTs in a neuroblastoma cell line [87], whereas truncation at Ile151 efficiently induced tau polymerization in vitro and in transgenic models of tauopathy [89]. Cleavage of tau inside the unstructured PD may thus represent an important mechanism to regulate its function.

### 4.3. Interactions of the Region Encoded by Tau Exon 4 with the Dynactin Complex

Another reported physiological role of the central region is facilitation of the MT binding to the dynactin complex. Presence of the region encoded by the short exon 4 is sufficient for binding of tau to the C-terminus of dynactin p150 [71]. Since the C-terminal domain of p150 forms an α-helical bundle [70], one can hypothesize that the only secondary structure motif encoded by exon 4, the transient helix Ser113–Val122, is the structural element responsible for interaction of this region of tau with the dynactin complex. As already discussed in Section 3.2, interactions of tau with p150 enhance binding of dynactin to MTs with a possible impact on the axonal transport.

### 4.4. Structural Properties of Variable Central Region of MAP2c

The N-terminal part of the central region of MAP2c consists of amino acids ${}_{119}$QPAALPL${}_{125}$ (encoded by the C-terminus of exon 6) with a strong polyproline II propensity and of the sequence encoded by short exon 7, including another polyproline II motif, a proline-rich region ${}_{134}$PPSPPPSP${}_{141}$. In the high-molecular weight MAP2 isoforms, the proline-rich region is followed by long inserts (encoded by exons 8–11 in MAP2a and 9–11 in MAP2b, see Figure 1). In MAP2c and MAP2d, the proline-rich region is directly connected to a flexible linker [57], followed by a sequence with a polyproline II propensity, and by a structural and functional motif not present in tau40 (α-helix flanked by polyproline II, shown in the yellow box in Figure 4).

### 4.5. Neural-Activity-Dependent Phosphorylation of MAP2c Epitope AP-18

The sequence ${}_{134}$PPSPPPSP${}_{141}$ matches a consensus motif PxSPxP (green box in Figure 4) recognized by proline-directed kinases (e.g., GSK3β). When phosphorylated at Ser136, the motif is specifically recognized by the antibody AP-18 [90]. The amino acid composition, resulting in very high propensity to adopt polyproline II conformation, and increased rigidity [57] document that ${}_{134}$PPSPPPSP${}_{141}$ is a well-defined structural motif in free MAP2c. Phosphorylation of Ser136 does not influence interactions of MAP2c with MTs, but relation to other biological functions has been reported. Greatly reduced phosphorylation of Ser136 was observed in the rat olfactory bulb after olfactory restrictions [91] and in rat hippocampus after behavioral training [92], suggesting association with contextual memory. Epinephrine increased phosphorylation of MAP2c in rat pheochromocytoma cells, most likely by activating $\alpha_{2}$-adrenoreceptor mediated, ERK/PKC-dependent signaling pathways, and a role in nerve cell differentiation was proposed [93]. However, it should be noted that molecular mechanisms of the mentioned effects are unknown and influence of other factors cannot be excluded. It is also possible that phosphorylation at Ser136, easily observable using the AP-18 antibody, was accompanied by phosphorylation at other sites that were not probed in the aforementioned studies.

### 4.6. Helical Motif of MAP2c Flanked by PKA Phosphorylation Sites and Involved in Interactions Interfering with MT Binding

The structural motif shown in the yellow box in Figure 4 contains one of the most populated transient helices in MAP2c, surrounded by polyproline II regions with multiple phosphorylation sites. Here we present an example of interactions controlling cytoskeletal dynamics and of their regulation by one particular kinase. The presence of arginines and lysines creates several consensus sites of Ser/Thr kinases recognizing R/KxxS/T and R/KR/KxS/T motifs (shown in red in Figure 4), such as PKA or protein kinase C. Real-time phosphorylation measurement revealed that Ser184 and Thr220 (red in Figure 4) are preferred targets of PKA in this region [40]. Interestingly, the rates of phosphorylations of Ser184 and Thr220 are comparable but Thr220 is dephosphorylated much faster than Ser184 by Ser/Thr phosphatases PP2b, PP2A${}_{c}$, and PP1A${}_{c}$, indicating that signaling may be also controlled by variations of dephosphorylation rate [94]. When Ser184 and Thr220 are phosphorylated, the flanking regions represent motifs recognized by the regulatory proteins 14-3-3 (phosphate group in an extended conformation [95]). Interactions of these motifs with 14-3-3ζ regulate ability of MAP2c to promote tubulin polymerization [40]. Furthermore, the transient α-helix between the PKA sites is one of the regions which show (together with MTBRs) signs of binding to non-canonical SH3 domain of plectin [57,96]. Plectin cross-links MTs with other cytoskeletal proteins and interferes with the MT-stabilizing function of MAPs [96]. The motif of MAP2c discussed in this subsection thus seems to influence MT dynamics in a complex manner, regulated by PKA phosphorylation. As this motif is present only in MAP2 isoforms, it may represent one of structural determinants distinguishing MAP2 from tau.

### 4.7. Summary

Segments between acidic N-terminal and basic proline-rich domains of tau40 and MAP2c differ substantially in structural propensities and functions. The central part of tau seems to be a region specifically targeted by proline-directed kinases and potentially influencing function of other regions of tau (regulation of MT dynamics). A unique proline-rich motif of MAP2c includes Ser136, phosphorylated in connection with the neural activity. MAP2c, but not tau40, contains a helical motif interacting with protein partners and surrounded by PKA phosphorylation sites regulating the interactions and consequently MT dynamics.

## 5. Proline-Rich Domains

The proline-rich regions P1 and P2 (brown boxes in Figure 1 and Figure 2) present in the middle of the tau40 and MAP2c sequences (tau exons 7/9, MAP2c exon 15) exhibit moderate sequence similarity. The proline-rich regions contain multiple SH3-binding (PxxP), phosphorylation (S/TP, R/KxxS/T), and lysine acetylation sites (Figure 5), contribute to the interactions with tubulin and actin [10,42,97], and are involved in intramolecular interactions with the N-terminal regions [38,57]. Structural features and their relation to biological functions are discussed together for proline-rich regions of tau40 and MAP2c in this section.

### 5.1. Structural Properties of Proline-Rich Domains of Tau and MAP2c

Amino-acid composition of the proline-rich regions determines their general physico-chemical properties: positive charge and tendency to form polyproline II structures. However, the charge distribution and secondary structure propensities are not uniform. The expected polyproline II conformation is most frequent, but the actual population of this structure varies along the sequence (Figure 2 and Figure 5). Segments with the highest populations of polyproline II structures exhibit increased rigidity in NMR relaxation experiments [38,57] (Figure 5), proving that formation of such motifs significantly affects behavior of free tau40 and MAP2c. A slight preference for α-helical conformation was observed only for residues 190–200 and 237–242 in tau40 (Figure 5). Phosphorylation can significantly alter the local conformation. Examples of such phosphorylation effects and their role in regulation of physiological functions of the observed specific structural motifs are discussed below.

### 5.2. Tubulin-Binding Motif in Tau Region P1

The first discussed structural motif influenced by phosphorylation is the tubulin-binding site in the P1 region of tau40 (Figure 5). Interactions with tubulin were observed for residues Arg170–Ser185, exhibiting the highest polyproline II propensity in the P1 region [41,42]. Phosphorylation of a short synthetic peptide derived from the tau P1 region (${}_{174}$KTPPAPKTPP${}_{183}$, green frame in Figure 5) further stabilized the polyproline II conformation [34]. It is not known if such phosphorylation-induced structural change plays a role in physiological function of the proline-rich domain, but relation of phosphorylation in the discussed motif to neurodegenerative diseases has been reported: phosphorylation of Thr175 in this region by GSK3β is associated with amyotrophic lateral sclerosis with cognitive impairment [98] and was recently observed also for traumatic brain injury [99]. Furthermore, the phosphomimetic mutation T175D increased GSK3β activity, resulting in phosphorylation of Thr231 [100] in another important structural motif, discussed in Section 5.4.

### 5.3. Phosphorylation-Controlled Conformational Switch in Tau Epitope AT8

The second motif discussed in this section is the sequence of tau shown in the pale green box in Figure 5. When phosphorylated, the sequence is recognized by the antibody AT8, which is used to determine progression of the Alzheimer’s disease *post mortem*. In free, unphosphorylated state, the motif does not prefer any secondary structure. However, phosphorylation at Ser202 and Thr205 induces formation of a helical turn, observable in NMR spectra [101]. Remarkably, this helical structure prevents formation of tau fibers. Phosphorylation of Thr205 mediated by p38γ also leads to disruption of the PSD-95–tau–Fyn complex responsible for Aβ toxicity [102]. Additional phosphorylation at Ser208 disrupts the turn and promotes tau aggregation in vitro [103]. The third phosphorylation seems to favor the polyproline II conformation, as the triply phosphorylated epitope binds to the AT8 antibody in this conformation [104]. This example illustrates how phosphorylation can completely revert local conformational behavior and alter physiological function of the given motif.

From the biological point of view, it is also interesting how is the sequential phosphorylation achieved in the cell. Ser202 and Thr205 can be phosphorylated by activated ERK2 [103], or by the action of CDK5 and GSK3β kinases, known to associate with tau and MTs in the brain [105]. Here we only describe the synergistic phosphorylation by CDK5 and GSK3β, as an example of *substrate priming* in multiple phosphorylation sites. In the normal adult brain, CDK5 activated by protein p35 (or p39) phosphorylates Ser199 and Ser202, and GSK3β is able to phosphorylate only Ser202. If GSK3β is up-regulated, it recognizes the ${}_{202}$pSPGT${}_{205}$ site created e.g., by p35/CDK5, and efficiently phosphorylates Thr205 [105,106]. Additional phosphorylation at Ser208 was achieved in vitro by addition of the rat brain extract [103].

Physiological role of the epitope AT8 is ambiguous. Phosphorylation of Ser202 and Thr205 contributes to the regulation of tau interactions with MTs, but it is not sufficient to inhibit MT binding [107]. Physiological role of the corresponding sequence of MAP2 isoforms is better described. The MAP2c site ${}_{256}$TPGTPGTPS${}_{264}$ closely resembles the AT8 sequence of tau (Figure 5) and seems to be phosphorylated similarly to tau [28]. The c-Jun N-terminal kinase-1 (JNK1) phosphorylates in vitro and in vivo all three threonines of rat MAP2a/b corresponding to tau Ser202, Thr205, and Ser208. In vivo experiments with phosphomimetic mutations of these threonines showed that their phosphorylations increase affinity of high-molecular weight MAP2 isoforms for MTs, stabilize MTs, and induce dendrite growth [108]. Therefore, phosphorylation of the discussed motif in MAP2 isoforms seems to be important for normal neuron morphology.

### 5.4. Formation of a Salt Bridge in Tau Epitope AT180 Interferes with MT Stabilization

The third example of a functionally important motif in the proline-rich region is the sequence of tau shown in the pale blue box in Figure 5. This site is particularly interesting because NMR line broadening was observed for its amino acids in the presence of tubulin [41,42] and mutagenesis confirmed a direct interaction [42]. However, this region of the tau40-tubulin complex is dynamic [42] and the corresponding electron density was not observed in a recent cryo-EM study of MT-tau interaction [43]. The interactions with MTs are strongly influenced by phosphorylation. Ser235 in the AT180 epitope is the most rapidly phosphorylated residue among the ERK2 sites in vitro [84] and it is also a target of several other kinases including p35/CDK5 or SAPK/p38 [85,105]. Phosphorylation of Thr231 in the same epitope occurs at normal physiological conditions. However, GSK3β phosphorylation of Thr231 is greatly facilitated (primed) by preceding p35/CDK5 phosphorylation of Ser235, in a similar manner as described for the AT8 epitope. The discussed site (pale blue box in Figure 5) phosphorylated at Thr231 and Ser235 is recognized by the antibody AT180 and has a greatly reduced ability to promote MT polymerization. Nuclear magnetic resonance data showed that the structural basis of this effect is formation of a salt bridge between phospho-Thr231 and Arg230, presumably competing with the formation of intermolecular salt bridges to tubulin [31]. Moreover, phosphorylation at Ser235 increases helical propensity of the AT180 epitope, and this trend is further enhanced by phosphorylation of Ser237 and Ser238 [31]. The shift of conformational equilibrium from polyproline II to helical structures does not affect MT binding [31], but may be important for interactions with SH3 domains because the discussed site overlaps with the Class I SH3-recognition motif ${}_{230}$RTPPKSP${}_{236}$ [109] (*vide infra*). The MAP2c site ${}_{289}$TPPKSPAT${}_{296}$ is also similar to the corresponding AT180 site of tau, but the penultimate serine is replaced by alanine (pale blue boxes in Figure 5).

### 5.5. Specific Phosphorylation of Ser214 by PKA and 14-3-3 Binding in the AT100 Epitope of Tau

The fourth discussed motif, shown in the yellow box in Figure 5, is an example of a site controlled by a very complex regulation network. It contains recognition sites of proline-directed kinases and is also efficiently phosphorylated by PKA. When phosphorylated at Thr212, Ser212, and Thr217, the site is recognized by the antibody AT100 [110]. The mentioned residues are not phosphorylated independently. Phosphorylation of Thr212 by GSK3β is activated by previous phosphorylation of the neighboring AT180 epitope, but inhibited by PKA phosphorylation of Ser214 (shown in red in Figure 5). Importantly, tau40 is phosphorylated by PKA at Ser214 much more rapidly than at Ser208 in vitro [111]. Furthermore, the PKA-phosphorylated site binds the regulatory proteins 14-3-3, which competes with MT binding [95,112,113,114,115]. Finally, the discussed site overlaps with the Class II SH3-recognition motif ${}_{216}$PTTPTR${}_{221}$ [109] (*vide infra*). Please note that the presence of the Class II motif increases population of polyproline II conformation, typical for complexes with both SH3 domains and 14-3-3 proteins.

In contrast to the AT8 and AT180 epitopes, the sequence of the tau epitope AT100 and the corresponding sequence in MAP2c (yellow boxes in Figure 5) differ, resulting in substantially different phosphorylation patterns and intermolecular interactions. The most rapidly phosphorylated target of PKA in the tau molecule, Ser 214 [111], aligns with Gly270 in MAP2c. Therefore, MAP2c is not efficiently phosphorylated by PKA anywhere in P2 [40]. Furthermore, the MAP2c sequence does not exhibit the strong polyproline II propensity of the corresponding tau motif. As a consequence, 14-3-3 protein, primarily recognizing phosphorylation sites with polyproline II conformation [116], binds to P2 of tau [95], but not of MAP2c [40] (Figure 5).

### 5.6. Binding Sites for the SH3 Domains

In addition to the interactions with MTs, the proline-rich domains specifically interact with SH3 domains of the Fyn kinase and of other proteins. Analysis of the populations of conformations of free tau40 and MAP2c helps to understand the structural basis of these interactions and differences between tau40 and MAP2c. Peptides bound to SH3 domains adopt polyproline II conformation [109]. Tau40 and MAP2c contain 7 and 13 minimal SH3 binding motifs PxxP, respectively, most of them in the P2 region. Among them, one classical Class II motif (${}_{216}$PTTPTR${}_{221}$) and one classical Class I motif (${}_{230}$RTPPKSP${}_{236}$) are present in tau and one Class I motif is present in MAP2c (${}_{288}$RTPPKSP${}_{294}$). Binding assays performed with synthetic biotinylated peptides showed that the Fyn SH3 domain binds preferentially to the Class II site of tau [109] and Class I site of MAP2c [117]. Remarkably, Class I and Class II motifs in free tau40 and MAP2c prefer the polyproline II conformation much more than the minimal SH3-binding motifs PxxP. This suggests that the higher affinity of the Class I and Class II motifs to the SH3 domains are not only due to the presence of the positive charge, but also due to the optimal backbone conformation, highly populated already in the free state.

Binding of SH3 domains to Class I and Class II motifs of tau and MAP2 isoforms is greatly reduced by phosphorylation [109,117]. In addition to introducing a negative charge, phosphorylation can also alter secondary structure propensity. We hypothesize that such conformational changes may distinguish the Class I sites of tau and MAP2c. Sequences of Class I motifs are identical in tau and MAP2c, but the following amino acids differ: slightly helical ${}_{237}$SSAKS${}_{241}$ in tau40 corresponds to ${}_{295}$ATPKQ${}_{299}$ with a strong polyproline II propensity in MAP2c (Figure 5). As mentioned above, the helical propensity around Lys240 in tau40 is greatly enhanced by phosphorylation of two serines missing in MAP2c (Ser237 and Ser 238). Such specific phosphorylation may selectively perturb the Class I SH3 binding site in tau, without affecting it in MAP2c.

### 5.7. Summary

The proline-rich domains of tau40 and MAP2c represent an important regulatory unit, controlled by multiple kinases and interacting proteins. Several differences in structural features, phosphorylation patterns, and molecular interactions of proline-rich domains of tau40 and MAP2c are likely to represent a basis for functional specificity of these proteins.

## 6. Microtubule-Binding Domains

Microtubule-binding domain (MTBD, violet boxes in Figure 1 and Figure 2) is the most thoroughly studied region of tau and MAP2 isoforms. It consists of up to four imperfect repeats (R1–R4, R2 is present in MAP2d but missing in MAP2c) of 31 or 32 amino acids (Figure 6). The sequence immediately following MTBR4 (R${}^{\prime }$) resembles the N-terminus of the aforementioned motif. As sequences, conformation, and dynamics of MTBDs of tau40 and MAP2c are highly similar [38,39,57], they are discussed together in this section.

### 6.1. Structural Properties of Microtubule-Binding Domains of tau40 and MAP2c

Microtubule-binding repeats in free tau40 and MAP2c contain the same motif, consisting of nine extended and relatively rigid residues, and of a stretch of 22 or 23 more flexible and highly conserved residues, which tends to form turns shown in Figure 6 [38,39,57]. The overall charge of the repeats is positive (Figure 2). The repeats are involved in normal physiological interactions with tubulin [38,41,42,43], plectin [57,96], actin [10], 14-3-3 [40,95,115,118], and form core structures of pathological tau filaments [24]. To discuss how are the described structural features of tau40 and MAP2c related to their physiological functions, we briefly review what is known about the conformation of tau in complexes with the most important binding partners, tubulin and actin.

### 6.2. Interactions with Microtubules

Nuclear magnetic resonance [38,41,42,119] and cryo-EM [43] data provided an insight into the specific interactions of tau with MTs. Residues of tau MTBD interacting with MTs and unpolymerized tubulin were identified based on broadening and shifts of peaks in the NMR spectra [38,41,42] (blue bars in Figure 6). Building an atomic-resolution model of the complex with MTs is complicated by presence of multiple potential binding sites on both partners. Due to the favorable time-scale of the interaction, it was possible to gain useful structural details from liquid-state NMR. Transferred nuclear Overhauser effect was recorded for a MT-bound tau fragment consisting of residues Lys268–Asn312, and used to identify hydrogen atoms closer than 0.6 nm in tau conformers bound to MTs [41]. Structural models calculated from the observed contacts converged in two regions folded to hairpin conformations. The hairpins consisted of turns formed by the conserved PGGG motifs and of extended hexapeptides identified previously [120] as the aggregation sites of the second and third MTBR (yellow boxes in Figure 6). Another binding model was derived from cryo-EM data [43]. Local resolution in complexes of MTs with synthetic tau constructs consisting of four identical copies of MTBRs 1 and 2 was sufficient to observe density corresponding to extended chains of tau residues but not to identify individual residues. Using Rosetta [121], the observed density was assigned to stretches of residues including the aforementioned hexapeptides found in extended conformation in the NMR model. The PGGG motifs were not modeled due to the lack of density, but the overall shape of the tau fragment in the cryo-EM model was inconsistent with formation of a hairpin. This discrepancy between cryo-EM and NMR data may reflect different binding modes of tau.

In the cryo-EM study [43], tau was found to bind to the outer surface of MTs along individual protofilaments, in the proximity of tubulin helices 11 and 12 and of the C-terminal tubulin tail. Earlier low-resolution cryo-EM study [122] and sequence homology suggest that MAP2c binds to the MTs in a similar manner. It has been also proposed that tau and MAP2 bind to MTs in a different manner, that MTBRs interact specifically with β-tubulin in the interior of MTs and proline-rich domains bind to the outer surface of MTs [1,123]. This model assumes that the PGGG sequence forms a loop which interacts with the taxol-binding site of β-tubulin, and is thus compatible with the NMR model. Both types of interactions with MTs (binding to the outer and inner surface) are supported by a solid experimental evidence: nanogold particles attached close to the PGGG motif were clearly observed inside [123] and outside [122] MTs. However, high-resolution data are available only for tau bound to the outer surface, despite of efforts to prepare samples with MTBRs interacting with the inner surface [43].

It is obvious that remarkable progress was achieved in characterization of tau-MT complexes, but detailed structures of MT-bound tau or MAP2 isoforms are not available yet. Nevertheless, known structural data already allow us to look for structural features observed in the complexes that are present already in the free state. In all structural models, the first nine amino acids of MTBRs are present in the extended conformation, which is also preferred in free tau40 and MAP2c. A correlation can be also seen between formation of turns in bound and free forms. Turns made by the PGGG motif were identified both in the structural ensembles selected by the ASTEROIDS analysis based on NMR data obtained for free tau40 and MAP2c [39,57], and in the structural models calculated from the contacts observed in NMR spectra observed in complexes of MTs with the tau fragments [41]. This turn was not observed in the Rosetta model based on cryo-EM data, but conformations of other residues are similar to the conformations favored in the ASTEROIDS ensembles. We can therefore conclude that the conformations adopted in the complex with MTs are often most populated in free tau40 and MAP2c.

Interactions with MTs are given not only by conformation of interacting residues, but also by their physical properties that can be regulated by post-translational modifications. For example, MT-stabilizing activity of both tau and MAP2 isoforms is inhibited by MARK phosphorylation [124,125], but phosphorylation by PKA inhibits the stabilizing activity of tau only [126,127]. Subtle differences in phosphorylation patterns and kinetics offer a possible explanation of observed differences. Serines in the middle of MTBRs are present in sequences representing recognition sites of several kinases, most notably the KxGS motif recognized by MARKs [128] (the complex overlapped motifs including the KxGS sites are presented in red boxes under the sequence in Figure 6). In general, PKA recognizes similar motifs. However, in vitro measurements of PKA phosphorylation kinetics revealed an important difference. PKA phosphorylates in vitro Ser324 in the third MTBR of tau40 (R3 in Figure 6) with a medium rate [111], but the in vitro PKA phosphorylation rate is negligible for all serines/threonines in MTBD of MAP2c [40]. Moreover, no serines or threonines are phosphorylated by PKA with a sufficient rate in the proline-rich domain of MAP2c [40], but Ser214 and Ser208 in the proline-rich domain of tau40 are residues of highest in vitro PKA phosphorylation rates [111]. It suggests that specific kinases can selectively control regulation of MT dynamics by tau vs. MAP2 isoforms: the same signal activating PKA can reduce interactions of MTs with tau, but not with MAP2.

### 6.3. Interactions with Actin and Other Proteins

Microtubule-binding domains of tau and MAP2c do not interact only with MTs, but are also involved in cross-linking and bundling individual actin filaments [11,129]. Liquid state NMR was used to describe interactions of tau40 with filamentous actin in detail [10]. In contrast to the mostly electrostatic interactions of the proline-rich domain [97], MTBD binds to the hydrophobic pocket between actin subdomains 1 and 3 on the surface of the actin filaments. Amino acids involved in the interactions are present in two helical (α or $3_{10}$) segments corresponding to residues 260–268 and 277–283. The former region forms a turn in both free and MT-bound tau40. Remarkably, the latter region is extended in a free state and in pathological tau filaments, and overlaps with the aggregation site (*vide infra*). The total number of interacting regions of either type is seven per tau40 molecule. A single helical binding region is sufficient to form a complex with F-actin, but two helical sites have to be present in order to bundle actin filaments. This explains how tau (and other MAPs) can cross-link actin filaments in the nerve cell. Similar to the MT binding, interactions with actin can be affected by post-translational modifications, but the effects may differ as the interactions are of a different nature. It has been reported for MAP2c that phosphorylation of the KxGS motifs favors localization of MAP2c in the actin cytoskeleton, and proposed that such colocalization may directly influence neurite outgrowth [130].

Other proteins described to interact with MTBD are isoforms of regulatory protein 14-3-3 [40,95,115,118]. Although 14-3-3 specifically binds phosphopeptides, it also electrostatically interacts with MTBDs of unphosphorylated tau40 and MAP2c [40,95]. The binding is further stabilized by phosphorylating tau40 and MAP2c in proline-rich and C-terminal regions [40,95,118]. Overlap of the interacting regions for 14-3-3 and for tubulin explains how 14-3-3 regulates interactions of tau40 and MAP2c with MTs. Another example of a protein interfering with MT binding is the cytolinker plectin, whose non-canonical SH3 domain interacts with MTBD and with the helical structure between the PKA sites Ser184 and Thr220 of MAP2c (yellow box in Figure 5), and thus competes with microtubules for MAP2c [57,96].

### 6.4. Aggregation of tau40

Despite its central role in normal physiology, MTBD of tau is primarily studied in connection with neuropathological changes involving tau. Stretches of six residues ${}_{275}$VQIINK${}_{280}$ and ${}_{306}$VQIVYK${}_{311}$ (yellow boxes in Figure 6) close to the N-termini of the second and third tau MTBRs, respectively, are able to initiate tau aggregation [120]. Although the actual toxicity of the aggregates and other pathological forms of tau remains to be determined, formation of paired helical filaments in brains is a hallmark of Alzheimer’s disease and other neuropathies. A 95-amino-acid fragment of tau40, derived from the PHF core and encompassing the third and fourth MTBR, easily forms filaments in vitro under physiological conditions without the need for other inducers of polymerization [131]. The aggregation also distinguishes tau40 from MAP2c. Although potential role of MAP2 isoforms in neuropathies cannot be excluded, it is clear that MAP2c does not form paired helical filaments or similar aggregates. Comparison of the MTBD sequences immediately suggests two possible causes of the difference: (i) lack of the second MTBR and (ii) difference in three amino acids in the aggregation site of the third MTBR (compare yellow and pale green boxes in Figure 6). Xie et al. investigated these factors systematically, using heparin to induce in vitro aggregation [26,132]. Judging from thioflavin T fluorescence and from amount of sarkosyl-insoluble high-molecular weight products, tau isoform with three MTBRs (0N3R, see Figure 1) aggregated to similar extent as the isoform 0N4R, albeit with a slower kinetics, whereas MAP2c formed very small amount of aggregates. However, replacement of two residues in the tau isoform 0N3R with the corresponding amino acids of MAP2c (double mutation Y310T, P312K, using tau40 numbering) completely abolished the aggregation, while the reciprocal mutation of MAP2c (T337Y, K339P) created a protein aggregating to the similar extent as tau 0N3R, and, furthermore, with much shorter lag-time [26]. Core structures of tau aggregates (paired helical filaments and straight filaments) reconstructed recently from cryo-EM images of samples isolated from brains of Alzheimer’s disease patients [24] show that the critical residues pack against Leu376 and His374 of filamentous tau40. NMR data provided additional information about transient interactions with largely disordered “fuzzy coat” regions surrounding the core [44]. These interactions involve P1 region, transient α-helices in C-terminal and central regions, and N-terminal region.

### 6.5. Summary

Microtubule-binding domain is most intimately related to the physiological roles of MAPs. Populations of conformations of free tau40 and MAP2c resemble the structures observed in the MT-bound state. Distinct conformations were observed in the complexes with actin, where tau was found to form helical structures. The variety of target sites for post-translational modifications is lower in MTBDs than in the proline-rich domains, but the impact of the modifications on the interactions is great. The MT binding is also controlled by interactions (also phosphorylation-dependent) with 14-3-3 proteins.

## 7. C-Terminal Regions

Regions of potential physiological importance are also located in the sequence between the end of MTBD and the C-terminus of tau40 and MAP2c (cyan boxes in Figure 1 and Figure 2). The overall sequence homology in the C-terminal region of tau40 and MAP2c is high, but the existing small differences have a great impact on the presence of phosphorylation, interaction, and cleavage sites and substantially contribute to the functional differences between tau40 and MAP2c.

### 7.1. Structural Properties of C-Terminal Regions of tau40 and MAP2c

The first 25 residues of the C-terminal regions of tau40 and MAP2c prefer polyproline II conformation, but the actual propensity differs between tau40 and MAP2c. The middle of the regions is slightly helical, followed by a more extended segment. The sequences of MAP2c and tau40 end by highly conserved segments with a strong α-helical propensity. Conformational analyses (evaluation of populations of structures with continuous stretches of four amino acids in α-helical conformation and estimation of secondary structure propensity from the chemical shift values) revealed more than 20% population of α-helix for both proteins [38,39,55,57]. The C-terminus is also involved in intermolecular interactions with MTBRs and the N-terminal regions [38,57], playing an important role in the “paper-clip” model of tau40 [62].

### 7.2. Muscarinic Receptor Activation and the PHF-1 Epitope of Tau

The C-terminal region seems to be responsible for activation of cholinergic receptors by extracellular tau. Full-length tau40 and a synthetic peptide ${}_{391}$EIVYKSPVVSGDTSPRH${}_{407}$ (red frame in Figure 7) were reported to interact with cholinergic muscarinic receptors M1 and M3, elevating Ca${}^{2+}$ concentration inside neurons. Based on this finding, it has been proposed that neurotoxic effects of tau released from damaged nerve cells are mediated by activating the M1 and M3 muscarinic receptors [133]. The segment of tau activating the M1 and M3 muscarinic receptors and the corresponding segment of MAP2c ${}_{417}$EIITQSPSRSSVASPRR${}_{433}$ (green frame in Figure 7) are less similar in sequence than the rest of the C-terminal domains. Tyr394 of tau, phosphorylated by c-Abl [36], is replaced by threonine in MAP2c, and the whole region is more positively charged in MAP2c (Figure 2). Also, the polyproline II propensity differs between tau and MAP2c. In the M1/M3 muscarinic receptor binding site of tau, it is limited to its N-terminal part [39], but it is observed in the C-terminal region of the motif in MAP2c [57]. These differences suggest that the activation of the M1 and M3 muscarinic receptors and consequent neurotoxic effects are specific features of tau, released to the extracellular space after cell death.

The C-terminal region of tau contains multiple phosphorylation sites, including S/TP motifs (Figure 7). Among them, Ser404 and Ser422 of tau are rapidly phosphorylated by ERK2 [84]. The M1/M3 muscarinic receptor binding site of tau overlaps with an important multiple phosphorylation site (yellow box in Figure 7), recognized by the antibody PHF-1 when Ser404 and Ser396 are phosphorylated. The Ser404, Ser400, and Ser396 residues of tau are phosphorylated subsequently in a similar manner as described for Ser235/Thr231 in P2, and with a similar impact on MT binding and filament formation [134]. Interestingly, tau interacts with the M1 and M3 muscarinic receptors only when the binding site is unphosphorylated [135]. The PHF-1 epitope thus seems to play a dual role in the development of Alzheimer’s disease, promoting aggregation in the phosphorylated state but requiring dephosphorylation prior to the muscarinic receptor activation.

### 7.3. Rapid Phosphorylation at Ser435 and 14-3-3 Binding of MAP2c

As mentioned above, C-terminus of the MAP2c motif shown in the green frame in Figure 7 differs from the corresponding sequence of tau by higher population of polyproline II conformation. Moreover, Ser435 in a close proximity is rapidly phosphorylated in vitro by PKA, while phosphorylation of the corresponding Ser409 in tau is slow. The extended structure and phosphorylation at Ser435 make PKA-phosphorylated MAP2c a better target of 14-3-3 proteins than tau. The differences in PKA phosphorylation and consequently in 14-3-3 binding suggest that PKA represents an important branch point in the signaling pathways. The list of major PKA phosphorylation sites of MAP2c (Ser184 and Thr220 flanking the helical motif preceding the proline-rich region and Ser435, discussed here) and of tau40 (S214 in the proline-rich region and S324 in MTBD) shows that PKA phosphorylates tau inside, but MAP2c outside proline-rich and MT-binding domains. Thus, the same signal (phosphorylation by PKA) has different downstream effects [40].

### 7.4. Protective Role of the C-Terminal Helix

It is known that C-terminal truncation by apoptotic caspases at Asp421 increases the rate of tau aggregation [136]. The “paper-clip” model of tau explains this observation by transient interactions of the C-terminal α-helix with MTBRs, protecting the aggregation sites. The inherent affinity of the C-terminal α-helix to MTBRs is observed also in filamentous tau, where the helix forms transient contacts with the cross-β core [44]. Interestingly, the last 33 amino acids of tau40 and MAP2c are highly similar, except for the caspase-3 recognition site of tau (${}_{418}$DMVD${}_{421}$), which aligns with a sequence ${}_{444}$NLLE${}_{447}$ of MAP2c. In fact, MAP2c does not contain any caspase-3 recognition motif DxxD and is not cleaved by this enzyme [137]. The aggregation-promoting caspase-3 cleavage of the C-terminal α-helix of tau40, but not of MAP2c, is another functionally important difference between these MAPs.

### 7.5. Summary

The C-terminal regions of MAP2c and tau40 consist of less homologous sequences rich in phosphorylation and interaction sites and of the highly homologous α-helical motif at the very terminus. In tau40, but not in MAP2c, the helix is cleaved off by caspase-3, which facilitates aggregation. Similar to the proline-rich regions, the phosphorylation/interaction segment between the C-terminus and MTBD seems to play important regulatory roles specific for MAP2 and tau isoforms.

## 8. Global Structural Features of MAP2c and tau40

Tau40 and MAP2c exhibit distinct structural features also at the level of tertiary structure. The overall shape of the tau40 and MAP2c molecules is mostly given by electrostatic interactions between acidic N-terminal and positively charged C-terminal domains. The structural effect of the intramolecular electrostatic interactions is formation of the bent “paper-clip” conformations [62], discussed above. The “paper-clip” model explains the functional links between distant regions of MAPs, such as effects of the N-terminal regions on MT binding, or phosphorylation of N-terminal tyrosines by the Fyn kinase interacting with the proline-rich domain of tau40 and MAP2c. Moreover, close contacts observed in the N-terminal region of MAP2c, comprising the proposed steroid-binding pocket and the interaction site for the regulatory RII subunit of PKA, indicate formation of a hydrophobic core missing in tau [57]. In addition to electrostatic and hydrophobic interactions, disulfide bonds can be formed between cysteins in the second and third MTBRs of tau and MAP2 isoforms containing all four MTBRs. On one hand, such intramolecular oxidation seems to prevent aggregation of tau40, on the other hand, intermolecular disulfide bridges promote fibrilization of tau isoforms containing three MTBRs [131,138,139].

At higher concentration, formation of antiparallel dimers is expected based on the charge distribution. Both types of structures have been observed in early studies of tau [140,141] and MAP2c [142]. In the cellular environment, the intramolecular contacts contribute to the delicate equilibrium of interactions related to MT dynamics [143], aggregation of tau [38], and interactions with other partners. The biological relevance of the formation of antiparallel dimers is less clear. Quantitative data [80,93,144,145,146,147] show that prenatal cytosolic concentrations of tau and MAP2c in adult neurons (5 μM–10 μM) [147] are low compared to the conditions when dimerization was significant in vitro. However, formation of dimers can be expected in regions with locally increased tau and MAP2c concentrations [130]. Electrostatic dimerization of tau and MAP2 isoforms was proposed as a molecular mechanism of MT bundling [8]. The ability to form the dimers is altered by phosphorylation. Proline-directed cdc2-like kinase, phosphorylating P2 and MTBD of tau, promotes tau dimerization [141], whereas PKA, phosphorylating outside P2 and MTBD of MAP2c, reduces intermolecular MAP2c interactions [57]. In the case of tau, anti-parallel dimerization due to the electrostatic interactions competes with formation of paired helical filaments stabilized mostly by hydrophobic interactions and able to form inter-strand disulfide bridges [138]. This additional complexity provides a more complete picture of the balance between normal and pathological physiology of tau, but also complicates interpretation of experimental data.

## 9. Conclusions

**Figure 8:**
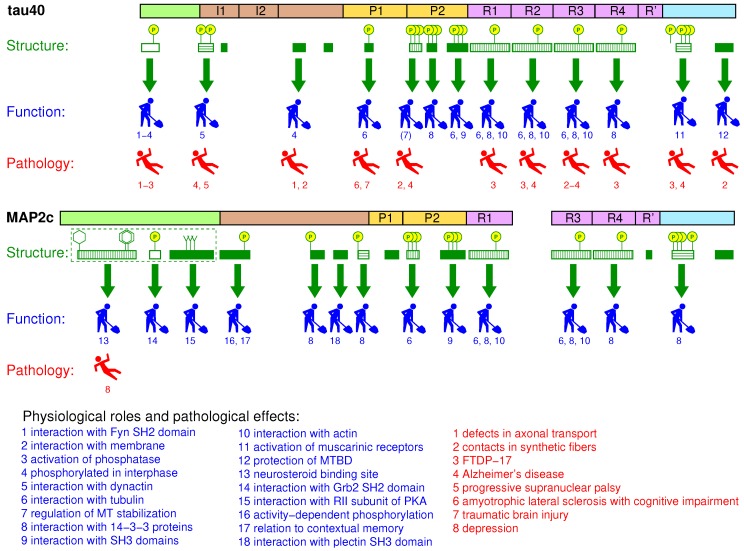
Association of biological functions with the transient structures of tau40 and MAP2c. Schematic representations of structural features (green symbols), normal physiological functions (blue symbols), and pathological effects (red symbols) are placed below horizontal bars showing regions of tau40 and MAP2c, colored as in Figure 2. The filled green rectangles represent all structural motifs with high population of transient secondary structures (continuous stretches of four residues found in polyproline II or α-helical conformation in more than 15% or 5%, respectively, structures in the ensemble selected by the ASTEROIDS analysis based on the experimental data), horizontal hatched green rectangles represent motifs associated with biological functions but with less populated secondary structures, vertical hatched green rectangles represent more complex motifs containing transient turn structures, and open green rectangles represent motifs not exhibiting significant secondary structure propensities but involved in long-range intramolecular interactions. The hexagons, Y-shapes, and circles with “P” above the rectangles indicate functionally important aromatic residues, hydrophobic aliphatic residues, and phosphorylation sites. The transient N-terminal compact structure of MAP2c is marked by the dashed frame. The thick green arrows show which structural motifs are associated with biological functions discussed in this paper, labeled by numbers explained under the diagrams.

A wealth of structural features revealed on tau40 and MAP2c proteins by state-of-the-art protein NMR, cryo-EM, and computations, summarized above, provided a detailed insight into molecular pathophysiology of an important class of MAPs. Tau40 and MAP2c are complex molecules and their physiological roles are yet not fully understood. The structure-function analysis is further complicated by the transient nature of the structural motifs of free forms of these MAPs. Nevertheless, the available data, discussed in detail in the preceding sections, already show a clear relation of numerous structural motifs of tau40 and MAP2c to various biological functions. The biological role is often manifested by specific interactions of short sequence motifs exhibiting transient, but clearly observable structural features. Such motifs can be therefore classified as molecular recognition elements of tau40 and MAP2c. Figure 8 summarizes structure-function relations discussed above and documents that most of the motifs with well-defined transient secondary structure can be associated with a particular function. Specific functions were also identified for more complex structural elements consisting of combination of several motifs (e.g., of extended and turn structures in MTBRs). Comparison of the transient local structures observed in free tau40 and MAP2c with the conformations in complexes with the binding partners (or in homologous complexes with other proteins sharing the same binding motif) often revealed conformational selection of structural motifs highly populated in the free state. However, conformational changes induced upon binding were also noticed (e.g., interactions of tau40 with actin and possibly of MAP2c with the RII subunit of PKA). Moreover, slower dynamics and presence of intramolecular contacts showed that biological functions also involve formation of compacted three-dimensional (“tertiary”) structures (e.g., N-terminal region of MAP2c or “paper-clip” structure of tau40). The physiologically important interactions of tau40 and MAP2c are further regulated by post-translational modifications, the specific phosphorylations and truncations discussed above represent illustrative examples.

The structure-function analysis also helps to understand molecular basis for the distinct biological activities of tau40 and MAP2c. Some of the differences are associated with structurally diverse N-terminal domains, not interacting directly with MTs. Other differences are more subtle, associated with local variation of amino-acid sequences of otherwise homologous C-terminal domains. The emerging picture of sensitive regulations of different tau and MAP2 functions by minor sequence changes, or slightly different phosphorylation kinetics, emphasizes intrinsic ability of disordered proteins to be specialized in interactions [148]. Structural principles observed site by site on related but functionally different tau40 and MAP2c may contribute new clues to decipher their physiological destiny as well as pathological role in chronic neurodegenerative diseases.

## Figures and Tables

**Table 1:** List of Ser/Thr kinases discussed in this review.

Kinase	Abbreviation	Proline-Directed	Activator
cAMP-dependent protein kinase	PKA	no	–
MT-affinity-regulating kinases	MARK1–4	no	–
extracellular signal-regulated kinase 2	ERK2	yes	MEK ${}^{a}$
glycogen-synthase kinase 3β	GSK3β	yes	–
cyclin dependent kinase 5	CDK5	yes	p35, p39
c-Jun N-terminal kinase 1	JNK1	yes	–
stress-activated protein kinase 4	SAPK4/p38δ	yes	–

${}^{a}$ MAPK/ERK kinase (also known as itogen-activated protein kinase kinase).

## Supplementary Materials

The following are available at https://www.mdpi.com/2218-273X/9/3/105/s1, Figure S1: Analysis of transient secondary structures of tau40, Figure S2: Analysis of transient turn structures of tau40, Figure S3: Analysis of transient secondary structures of MAP2c, Figure S4: Analysis of transient β-turn structures of MAP2c, Figure S5: Comparison of secondary chemical shifts of full-length MAP2c and of its fragments, Figure S6: NMR titration of dehydroepiandrosterone by MAP2c and its fragments.
