# Supplementary material for: Structure and Functions of Microtubule Associated Proteins Tau and MAP2c: Similarities and Differences
Source: Biomolecules. 2019 Mar 16;9(3):105. doi: 10.3390/biom9030105 (PMC6468450; doi:10.3390/biom9030105)
Supplement: Supplementary file 1 [file biomolecules-09-00105-s001.pdf]

biomolecules

MDPI

Review

Structure and Functions of Microtubule Associated Proteins Tau and MAP2c: Similarities and Differences

SUPPLEMENTARY MATERIALS

Kateřina Melková[1,2†], Vojtěch Zapletal[1,2†], Subhash Narasimhan[1†], Séverine Jansen[1,2], Jozef Hritz[1], Rostislav Škrabana[3,4], Markus Zweckstetter[5,6], Malene Ringkjøbing Jensen[7], Martin Blackledge[7] and Lukáš Žídek[1,2*]

1 Masaryk University, Central European Institute of Technology, Kamenice 5, 625 00 Brno, Czech Republic; katerina.melkova@ceitec.muni.cz (K.M.); vojtech.zapletal@ceitec.muni.cz (V.Z.); subhash.narasimhan@ceitec.muni.cz (S.N.); severine@chemi.muni.cz (S.J.), jozef.hritz@ceitec.muni.cz (J.H.); lzidek@chemi.muni.cz
2 Masaryk University, Faculty of Science, National Centre for Biomolecular Research, Kamenice 5, 625 00 Brno, Czech Republic
3 Institute of Neuroimmunology, Slovak Academy of Sciences, Dubravska cesta 9, 845 10 Bratislava, Slovakia; rostislav.skrabana@savba.sk
4 Axon Neuroscience R&D Services SE, Dvorakovo nabrezie 10, 811 02 Bratislava, Slovakia
5 Department of NMR-based Structural Biology, Max Planck Institute for Biophysical Chemistry, Am Fassberg 11, 37077 Göttingen, Germany; Markus.Zweckstetter@dzne.de
6 German Center for Neurodegenerative Diseases (DZNE), Von-Siebold-Str. 3a, 37075 Göttingen, Germany
7 University Grenoble Alps, CEA, CNRS, 38000 Grenoble, France; malene.ringkjobing-jensen@ibs.fr (M.R.J.); martin.blackledge@ibs.fr (M.B.)
* Correspondence: lzidek@chemi.muni.cz; Tel.: +420-549-498-393
† These authors contributed equally to this work.

CONTENT:

Figure S1: Analysis of transient secondary structures of tau40 [1]
Figure S2: Analysis of transient turn structures of tau40
Figure S3: Analysis of transient secondary structures of MAP2c [2]
Figure S4: Analysis of transient β-turn structures of MAP2c
Figure S5: Comparison of secondary chemical shifts of full-length MAP2c and of its fragments
Figure S6: NMR titration of dehydroepiandrosterone by MAP2c and its fragments

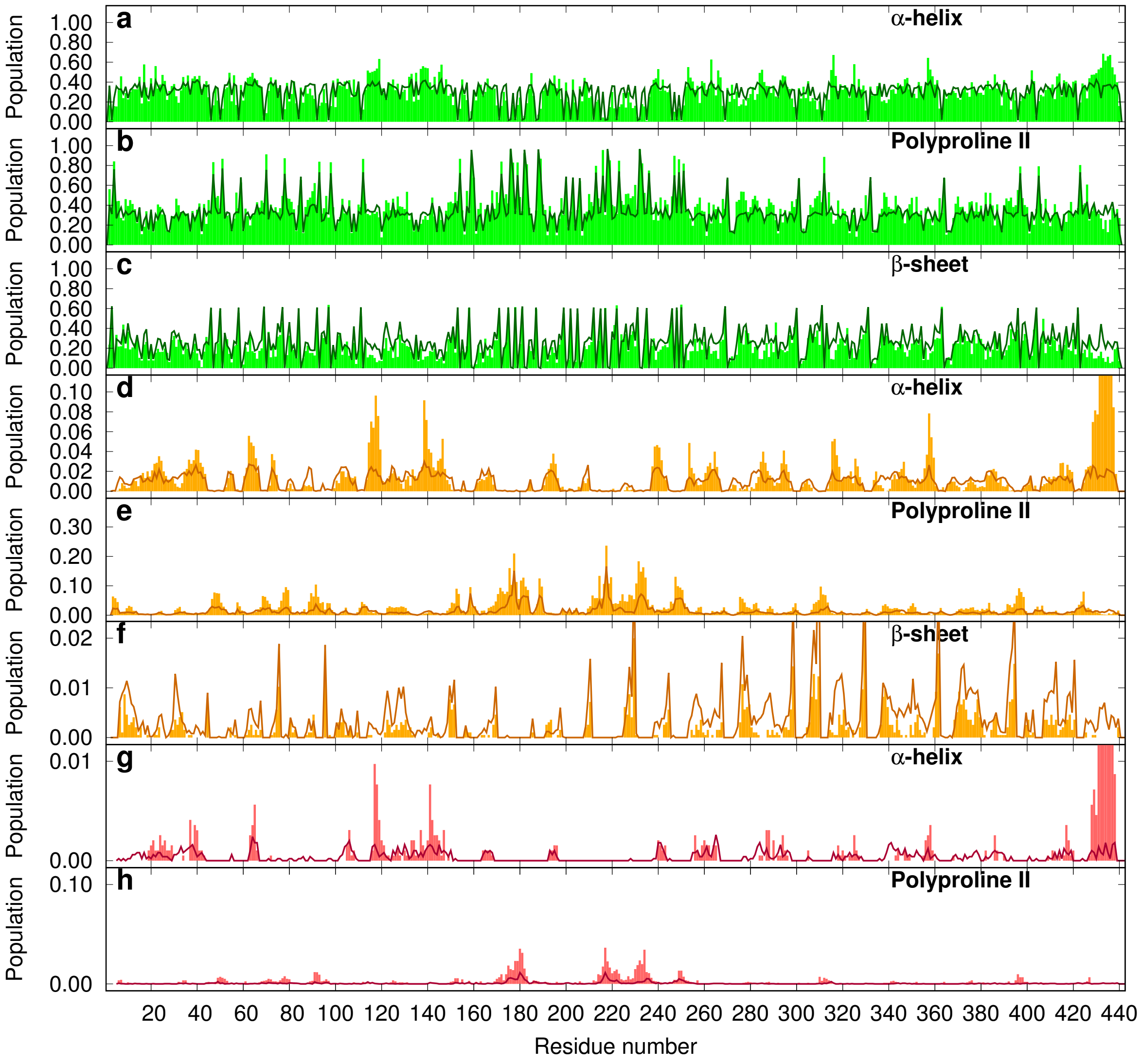

Figure S1. Populations of torsion angles in the regions of the Ramachandran plot corresponding to the (a,d,g) helical, (b,e,h) polyproline-II, and (c,f) *β*-sheet conformations of tau40 [1]. Populations of individual residues (a–c) and of continuous stretches of four (d–f) and seven amino acids in the given conformation in the ensembles of 600 structures selected by the ASTEROIDS analysis to fit the experimental data are shown as green, orange, and red bars, respectively. The orange and red bars are placed in the middle of the stretches. Populations in the original pool of structures (prior to ASTEROIDS selection) with statistical distribution of torsion angles, which are plotted as solid dark green, dark orange, and dark red lines, respectively.

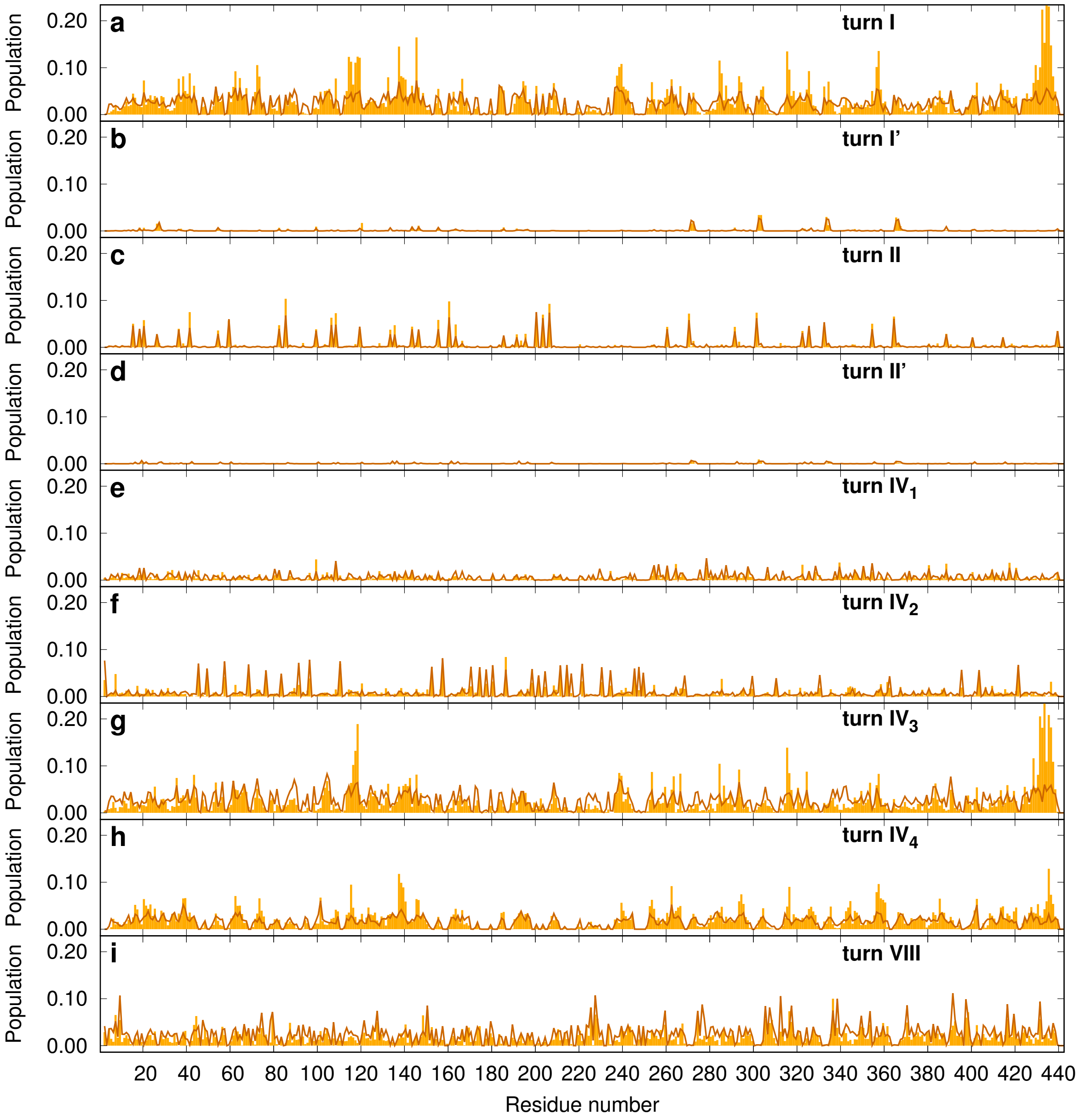

Figure S2. Populations of combinations of torsion angles in continuous stretches of four amino acids of tau40 corresponding to conformations in β turns classified according to de Brevern [3]. The orange bars and dark orange lines, placed in the middle of the turns, represent populations in the ensembles selected by the ASTEROIDS analysis and in the original pool of structures (prior to ASTEROIDS selection) with statistical distribution of torsion angles, respectively.

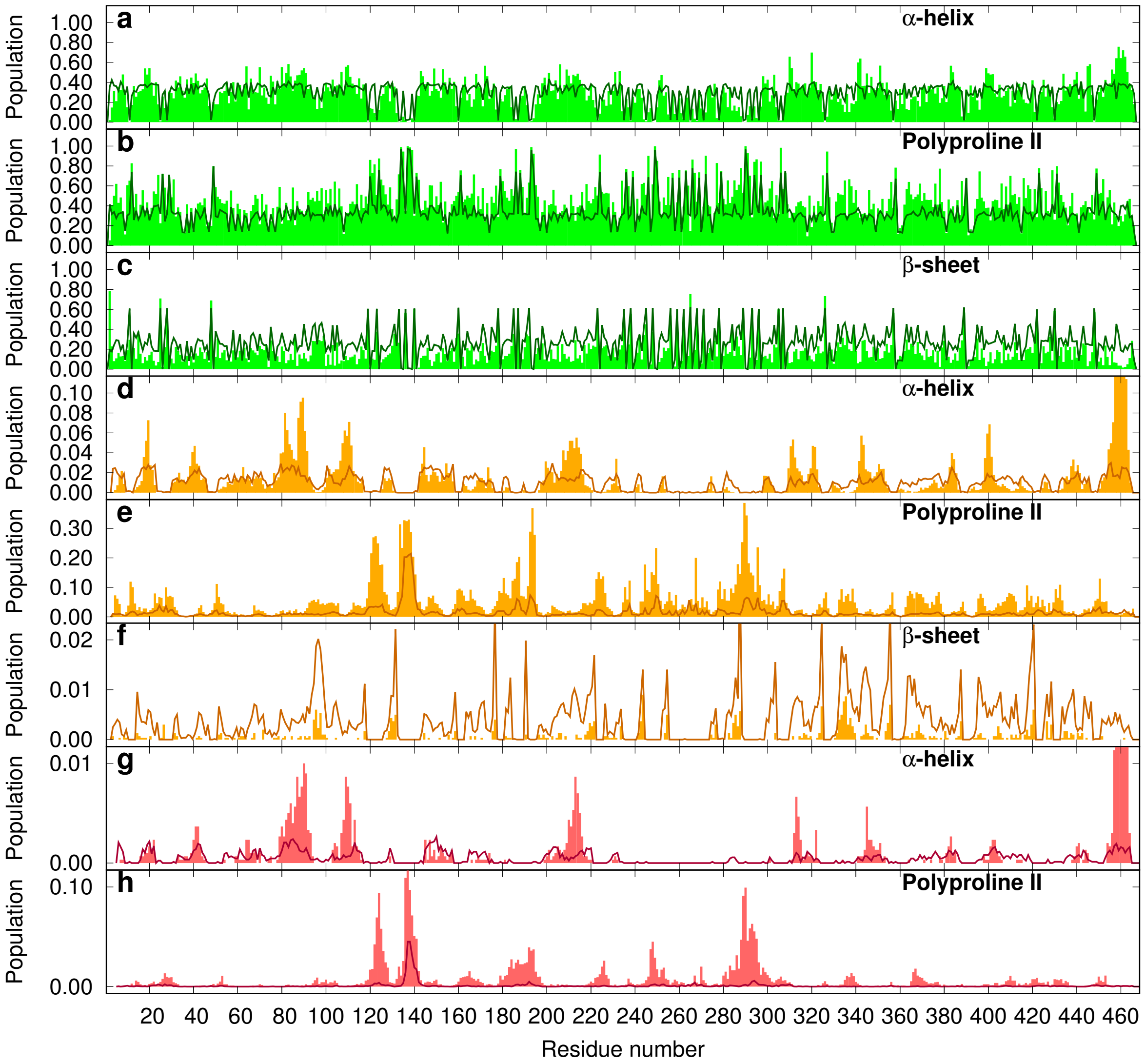

Figure S3. Populations of torsion angles in the regions of the Ramachandran plot corresponding to the (a,d,g) helical, (b,e,h) polyproline-II, and (c,f) β-sheet conformations of MAP2c [2]. Populations of individual residues (a–c) and of continuous stretches of four (d–f) and seven amino acids in the given conformation in the ensembles of 600 structures selected by the ASTEROIDS analysis to fit the experimental data are shown as green, orange, and red bars, respectively. The orange and red bars are placed in the middle of the stretches. Populations in the original pool of structures (prior to ASTEROIDS selection) with statistical distribution of torsion angles, which are plotted as solid dark green, dark orange, and dark red lines, respectively.

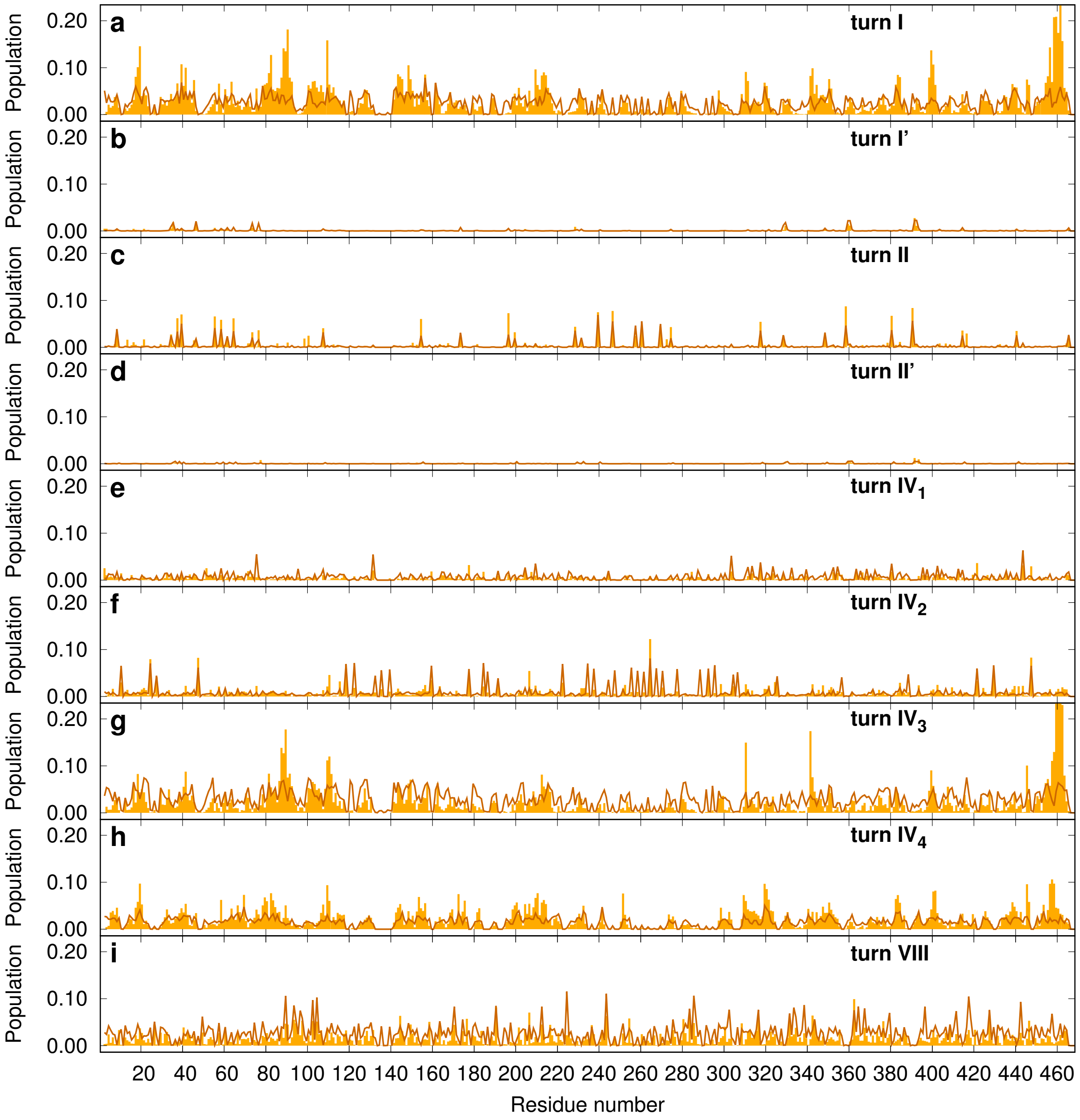

Figure S4. Populations of combinations of torsion angles in continuous stretches of four amino acids of MAP2c corresponding to conformations in β turns classified according to de Brevern [3]. The orange bars and dark orange lines, placed in the middle of the turns, represent populations in the ensembles selected by the ASTEROIDS analysis and in the original pool of structures (prior to ASTEROIDS selection) with statistical distribution of torsion angles, respectively.

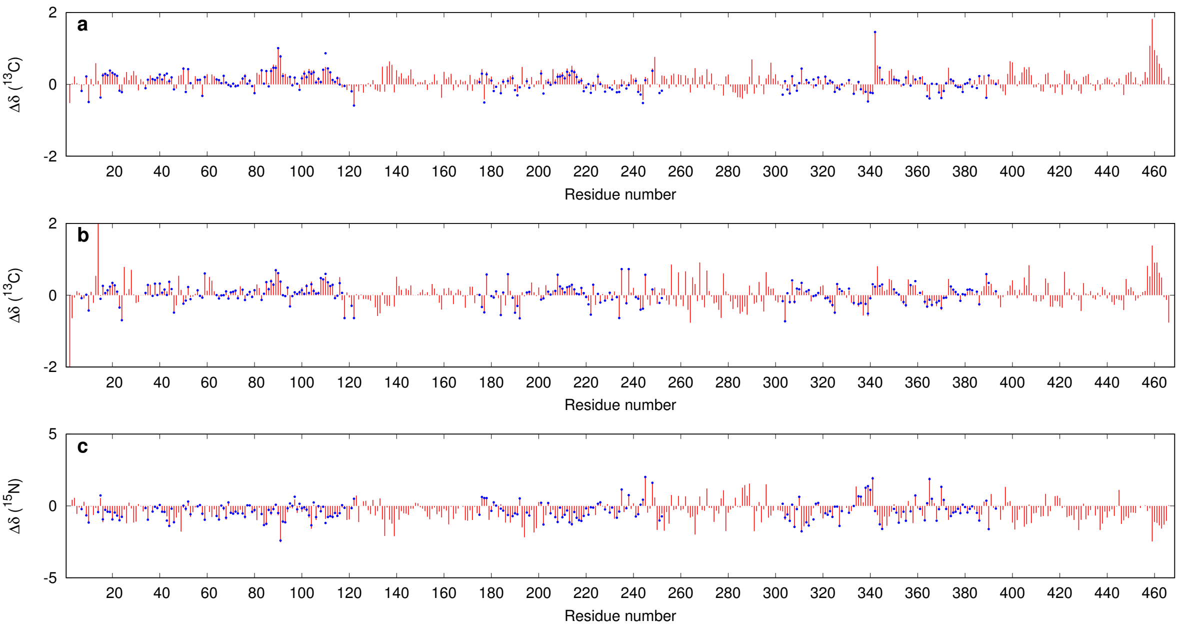

Figure S5. Secondary chemical shifts of $^{13}C^{\alpha}$ (a), carbonyl ^{13}C (b), and backbone amide ^{15}N (c) in full-length MAP2c [2,4] and MAP2c fragments consisting of residues 1–126, 159–254, and 300–399, assigned using the CACONCACO 5D NMR experiment [5]. Values of the full-length protein and of the fragments are shown as red bars and blue dots, respectively.

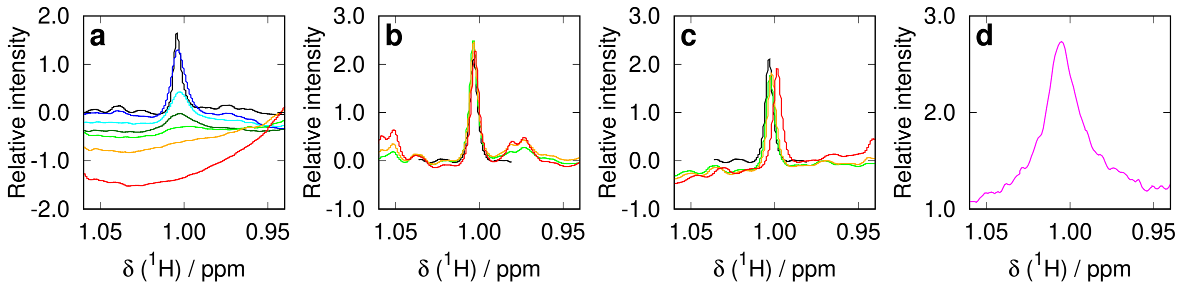

Figure S6. NMR titration of 200 μM dehydroepiandrosterone (obtained from MAPREG, Le Kremlin-Bicêtre, France) dissolved in 5% methanol and 0.2% Tween) by MAP2c (a) and MAP2c fragments consisting of residues 1–159 (b) and 159–467 (c). The colors of spectra indicate concentration of MAP2c or its fragment (black, 0 μM; blue, 3 μM; cyan, 6μM; dark green, 12 μM; light green, 25 μM; orange, 50 μM; and red, 100 μM). The sharp peak of methyl 20 (1.0 ppm) of free dehydroepiandrosterone broadens due to the interaction with MAP2c only in the presence of the full-length protein. Saturation transfer difference spectrum for selectively irradiated proton ζ2 of Trp14 in full-length MAP2c (d) proves that dehydroepiandrosterone binds to the proximity of Trp14 in the N-terminal region of MAP2c.

References

1. Schwalbe, M.; Ozenne, V.; Bibow, S.; Jaremko, M.; Jaremko, L.; Gajda, M.; Jensen, M.R.; Biernat, J.; Becker, S.; Mandelkow, E.; Zweckstetter, M.; Blackledge, M. Predictive Atomic Resolution Descriptions of Intrinsically Disordered hTau40 and α-Synuclein in Solution from NMR and Small Angle Scattering. *Structure* **2014**, *22*, 238–249.
2. Melková, K.; Zapletal, V.; Jansen, S.; Nomilner, E.; Zachrdla, M.; Hritz, J.; Nováček, J.; Zweckstetter, M.; Jensen, M.R.; Blackledge, M.; Žídek, L. Functionally specific binding regions of microtubule-associated protein 2c exhibit distinct conformations and dynamics. *J. Biol. Chem.* **2018**, *293*, 13297–13309.
3. de Brevern, A.G. Extension of the classical classification of β-turns. *Sci. Rep.* **2016**, *6*, 33191.
4. Jansen, S.; Melková, K.; Trošanová, Z.; Hanáková, K.; Zachrdla, M.; Nováček, J.; Župa, E.; Zdráhal, Z.; Hritz, J.; Žídek, L. Quantitative mapping of microtubule-associated protein 2c (MAP2c) phosphorylation and regulatory protein 14-3-3ζ-binding sites reveals key differences between MAP2c and its homolog Tau. *J. Biol. Chem.* **2017**, *292*, 6715–6727.
5. Nováček, J.; Janda, L.; Dopitová, R.; Žídek, L.; Sklenář, V. Efficient protocol for backbone and side-chain assignments of large, intrinsically disordered proteins: transient secondary structure analysis of 49.2 kDa microtubule associated protein 2c. *J. Biomol. NMR* **2013**, *56*, 291–301.

© 2019 by the authors. Licensee MDPI, Basel, Switzerland. This article is an open access article distributed under the terms and conditions of the Creative Commons Attribution (CC BY) license (http://creativecommons.org/licenses/by/4.0/).
